# Preparation of Tri(alkenyl)functional Open-Cage Silsesquioxanes as Specific Polymer Modifiers

**DOI:** 10.3390/polym12051063

**Published:** 2020-05-06

**Authors:** Katarzyna Mituła, Michał Dutkiewicz, Julia Duszczak, Monika Rzonsowska, Beata Dudziec

**Affiliations:** 1 Faculty of Chemistry, Adam Mickiewicz University in Poznan, Uniwersytetu Poznańskiego 8, 61-614 Poznan, Poland; katarzyna.mitula@gmail.com (K.M.); julia.duszczak@amu.edu.pl (J.D.); m.rzonsowska@gmail.com (M.R.); 2 Centre for Advanced Technologies, Adam Mickiewicz University in Poznan, Uniwersytetu Poznańskiego 10, 61-614 Poznan, Poland; midu@amu.edu.pl; 3 Adam Mickiewicz University Foundation, Rubiez 46, 61-612 Poznan, Poland

**Keywords:** open-cage silsesquioxanes, hydrolytic condensation, thermal analysis

## Abstract

The scientific reports on polyhedral oligomeric silsesquioxanes are mostly focused on the formation of completely condensed T_8_ cubic type structures and recently so-called double-decker derivatives. Herein, we report on efficient synthetic routes leading to trifunctionalized, open-cage silsesquioxanes with alkenyl groups of varying chain lengths from -vinyl to -dec-9-enyl and two types of inert groups (iBu, Ph) at the silsesquioxane core. The presented methodology was focused on hydrolytic condensation reaction and it enabled obtaining titled compounds with high yields and purity. A parallel synthetic methodology that was based on the hydrosilylation reaction was also studied. Additionally, a thorough characterization of the obtained compounds was performed, also in terms of their thermal stability, melting and crystallization temperatures (TGA and DSC) in order to show the changes in the abovementioned parameters dependent on the type of reactive as well as inert groups at Si-O-Si core. The presence of unsaturated alkenyl groups has a profound impact on the application potential of these systems, i.e., as modifiers or comonomers for copolymerization reaction.

## 1. Introduction

Organosilicon compounds are of great interest and they have been investigated during the past few decades, which is reflected in the growing number of papers and patents published each year. These systems offer development of methodologies for the formation of advanced, multifunctional materials due to the combination of organic and inorganic segments. One of the fundamental examples of organosilicon derivatives of magnitude importance is silsesquioxane (SQ). They are a worldwide known family of nanosized hybrid compounds of the general [RSiO_3/2_]_n_ formula, being represented by a versatile group of three-dimensional species where distinct structures may be formed, i.e., random, ladder, cage and incompletely condensed cage [1,2]. The most popular members of this family are cage silsesquioxanes, especially of cubic T_8_ type that may contain from one to eight functional/reactive groups (FG). The inorganic core is based on the Si-O-Si framework possessing a silicon atom at each vertex, being responsible for, i.e., the thermal stability of these systems. Reactive or inert moieties tetrahedrally coordinated at the silicon vertices affect SQs physical and chemical properties and result in their vast potential application. The studies on silsesquioxane based materials influenced almost every branch of science (i.e., medicine and biochemistry, material chemistry, optoelectronics, etc.), due to their structural diversity and interesting properties, i.e., thermal stability, oxidation resistance, dielectric coefficient, low toxicity, and possibility for their not complicated structural and functional modification [2,3]. As a consequence, these systems are promising candidates for building blocks for hybrid materials. Most studies concern the application of completely condensed silsesquioxanes, either mono- or octasubstituted T_8_ or, recently, the double-decker types [4,5,6,7]. On the other hand, open-cage silsesquioxanes, i.e., not-completely condensed systems have recently attracted much attention. The silsesquioxane trisilanol precursors enable the introduction of three reactive substituents to the Si-O-Si core by means of stoichiometric (hydrolytic condensation), but also catalytic (e.g., hydrosilylation, arylation, *O*-silylation), processes using functional groups that are already attached to the SQ core [8,9,10,11,12,13,14,15,16]. The symmetry of their trifunctional T_7_ open-cage derivatives is changed due to the lack of one vertex in the structure, which affects their physical properties (i.e., changes in molecular packing, melting points) [9,17,18,19,20]. However, they offer interesting synthetic possibilities due to the opportunity to introduce more than one functional group within the silsesquioxanes core bearing at the same time also inert ones. As a result, the perspectives for the further development of this group of compounds are vast, from nanostructured coating films [13], silica-grafted catalysts [21,22], tripodal ligands for transition metals [23], novel cage host molecules [24], aggregates in cosmetics and food manufacturing [25,26], or dentistry [27,28] to polymers modifiers as cross-linking agents [29,30,31,32,33,34]. Multi-functional SQs may additionally offer the possibility to obtain three-dimensional (3D) copolymeric networks, leading to substantial improvement in their physicochemical properties, i.e., increased thermal and oxidation resistance, mechanical properties, e.g., hardness, as well as the reduction of flammability, heat evolution, viscosity during processing, etc. [35,36,37,38,39,40,41,42,43,44,45,46,47].

This study was undertaken to design and elaborate efficient synthetic methodology for tri(alkenyl)functional silsesquioxane derivatives. Simple condensation reactions of chlorosilanes with silsesquioxane trisilanols allowed for us to obtain diverse types of incompletely condensed POSS compounds with three functional groups varying from -vinyl to -dec-9-enyl. The resulting tri(alkenyl)functional silsesquioxanes were obtained with high yields and were thoroughly analyzed spectroscopically. Their thermal stability, melting, and crystallization temperatures were evaluated at details (TGA and DSC) to tend to changes in thermal properties in relation with the reactive (FG) as well as inert (R) groups at Si-O-Si core. In parallel, we also studied a complementary synthetic protocol, while using hydrosilylation reaction of dienes with respective SQs bearing three Si-H reactive moieties. Unfortunately, the use of this method for the synthesis of a discussed group of unsaturated SQ derivatives was found to be unselective and it led to a mixture of products. As aforementioned, tri(alkenyl)functional silsesquioxane derivatives may be valuable multifunctional Si-O-Si based structural motifs for further modifications, e.g., as cross-linking agents for nanocomposite materials.

## 2. Materials and Methods

### 2.1. Materials

The chemicals were purchased from the following sources: chlorodimethylsilane (Sigma-Aldrich, Saint Louis, MO, USA, CAS: 1066-35-9, assay 98%), chloro(dimethyl)vinylsilane (Sigma-Aldrich, Saint Louis, MO, USA CAS: 1719-58-0, assay 97%), allyl(chloro)dimethylsilane (Sigma-Aldrich, Saint Louis, MO, USA CAS: 4028-23-3, assay 97%), 1,5-hexadiene (CAS: 592-42-7, assay 97%), Karstedt’s catalyst (platinum(0)-1,3-divinyl-1,1,3,3-tetramethyldisiloxane complex [Pt_2_(dvs)_3_] solution in xylene with 2% of Pt, Sigma-Aldrich, Saint Louis, MO, USA CAS: 68478-92-2), triethylamine (CAS: 121-44-8, assay ≥ 99.5%), calcium hydride (CAS: 7789-78-8, assay 98%), sodium (Sigma-Aldrich, Saint Louis, MO, USA CAS: 7440-23-5, cubes, contains mineral oil, assay 99.9%), benzophenone (Acros Organics, Geel – Belgium CAS: 119-61-9, assay 99%), 1,9-decadiene (TCI, Portland, OR, USA, CAS: 1647-16-1, assay 98%), R_7_(Si_7_O_9_)(OH)_3_ (Hybrid Plastics, Hattiesburg, MS, USA, SQ-R-OH; R = iBu CAS: 307531-92-6, Ph CAS: 444315-26-8). Toluene, tetrahydrofuran (THF), methanol, n-hexane, dichloromethane (DCM), chloroform-d, molecular sieves type 4Å from P.O.Ch Gliwice, Poland. Silica gel-MN-Kieselgel 60 from Fluka Chemie AG, Buchs, Switzerland. Chloro(hex-5-enyl)silane and chloro(dec-9-enyl)silane were obtained via the procedure described earlier [48]. SQ-iBu-OH was prepared prior to use, i.e., dissolved in DCM, precipitated in MeOH, and then dried under vacuum. All of the syntheses were conducted under argon atmosphere while using standard Schlenk-line and vacuum techniques. Toluene and n-hexane were dried over CaH_2_ and THF over Na with benzophenone before use and stored under argon over type 4Å molecular sieves. Amine was dried over CaH_2_ before use and then stored under argon in Schlenk.

### 2.2. Measurements

*Nuclear Magnetic Resonance* (NMR) measurements were conducted using: for ^1^H NMR-Bruker Ultrashield 300 MHz, for ^29^Si and ^13^C NMR—Brucker Ultrashield 400 MHz, using CDCl_3_ as a solvent. Chemical shifts are reported in ppm with reference to the residual solvent (CHCl_3_) peaks for ^1^H and ^13^C and to TMS for ^29^Si.

*Fourier Transform-Infrared* (FT-IR) spectra were recorded on a Nicolet iS5 (Thermo Scientific, Waltham, MA, USA) spectrophotometer that was equipped with a diamond ATR unit (iD7 ATR Optical Base). In all cases, 16 scans at a resolution of 2 cm^−1^ were collected, in order to record the spectra in a range of 4000−400 cm^−1^.

*Gas Chromatography* (GC) analysis was performed on Bruker 430 gas chromatograph that was equipped with a TCD detector and capillary column megabore HP-130 m (Hewlett Packard, Palo Alto, CA, USA).

*Thermogravimetric analysis* (TGA) was performed on a TGA Q50 (TA Instruments, New Castle, DE, USA) apparatus. All experiments were carried out in air atmosphere (flow rate 60 mL/min.) in a temperature range from RT to 1000 °C and at the heating rate of 10 °C/min.

*Differential Scanning Calorimetry* (DSC) analysis was carried out with the use of a DSC1 calorimeter (Mettler-Toledo). The samples of ca. 10 mg were heated in sealed aluminum crucibles in the temperature range of 0 to 200 °C with a heating rate of 10 °C min.^−1^ in nitrogen (25 mL min.^−1^). The glass transition temperature (T_g_) was designated as the inflection point in the curves that were obtained from the second run.

*Matrix-Assisted ultraviolet Laser Desorption/Ionization Time-Of-Flight Mass Spectroscopy* (MALDI-TOF-MS) were recorded on an UltrafleXtreme mass spectrometer (Bruker Daltonics), equipped with a SmartBeam II laser (355 nm) in 500–4000 m/z range. 2, 5-Dihydroxybenzoic acid (DHB, Bruker Daltonics, Bremen, Germany) served as matrix and it was prepared in TA30 solvent (30:70 v/v acetonitrile: 0.1% TFA in water) at a concentration of 20 mg/mL. The studied samples were dissolved in dichloromethane (2 mg/mL) and then mixed in a ratio 1:1 v/v with a matrix solution. Matrix/sample mixtures (1 µL) were spotted onto the MALDI target and then dried in air. Mass spectra were measured in reflection mode. The data were analyzed while using the software provided with the Ultraflex instrument—FlexAnalysis (version 3.4). Mass calibration (cubic calibration based on five to seven points) was performed using external standards (Peptide Calibration Standard).

### 2.3. Synthetic Procedures for Preparation of SQ-R-SiH R = iBu, Ph

The exemplary procedure is presented for the **SQ-Ph-SiH** compound and it is analogous for **SQ-iBu-SiH**. Into a two-neck round-bottom flask that was equipped with a magnetic stirrer, purged with argon and then placed in ice-water bath **SQ-Ph-OH** (1.03 g, 1.101 mmol), anhydrous THF (10 mL) and chloro(dimethyl)silane (0.39 mL, 3.4 mmol) were introduced and followed by dropping of Et_3_N (0.48 mL, 3.47 mmol) to the obtained solution. The reaction was carried out for 24 h at room temperature. Subsequently, insoluble solid of triethylammonium chloride was removed by filtration on a glass frit, and then volatiles and THF were eliminated via rotary evaporation. The crude product was dissolved in DCM and crystallized in cold MeOH. After methanol removal, the solid was dried in a vacuum. For the spectroscopic analysis please see Supplementary Materials (Section 1.1).

### 2.4. General Procedure for Preparation of tri(alkenyl)functional SQs (SQ-R-FG; R = iBu, Ph; FG = Vi, All, Hex, Dec)

#### 2.4.1. Procedure for Preparation of SQ-R-FG (R = iBu, Ph; FG = Vi, All, Hex, Dec) Compounds via a Hydrolytic Condensation Reaction

The exemplary procedure is presented for **SQ-iBu-Vi**. Trisilanol **SQ-iBu-OH** (0.31 g, 0.39 mmol), anhydrous THF (4 mL), and chloro(dimethyl)vinylsilane (0.17 mL, 1.21 mmol) were introduced into a two-neck round-bottom flask that was equipped with a magnetic stirrer and placed in an ice-water bath (the respective chloro(dimethyl)(hex-3-enyl)silane and chloro(dimethyl)(dec-5-enyl)silane were prepared according to the literature procedure [48]). This was followed by adding Et_3_N (0.17 mL, 1.23 mmol) to the reaction mixture. The reaction was carried out for 24 h at room temperature. Afterwards, insoluble solid of triethylammonium chloride was removed by filtration on a glass frit, and then volatiles and THF were eliminated via rotary evaporation. The crude product was washed several times with cold MeOH. After methanol removal, the solid was dried in a vacuum. In the case of **SQ-iBu-Hex** and **SQ-iBu-Dec**, 0.25 g (0.317 mmol) of **SQ-iBu-OH** and 0.4 mL anhydrous THF was used.

#### 2.4.2. Procedure for the Hydrosilylation of Dienes by SQ-R-SiH (R = iBu, Ph; diene = 1,5-hexadiene, 1,9-decadiene)

The exemplary procedure is presented for **SQ-iBu-Dec** and it is analogous for **SQ-R-FG** (R = Ph; FG = Hex, Dec). **SQ-iBu-SiH** (0.16 g, 0.166 mmol), anhydrous toluene (4.15 mL), and 1,9-decadiene (0.11 mL, 0.60 mmol) were added into a two-neck round-bottom flask equipped with a magnetic stirrer and a reflux condenser. This was followed by adding Karstedt’s catalyst (0.57 µL, 4.99 x10^−8^ mol) to the reaction mixture. The reaction was heated to 95 °C and then carried out to the complete Si-H bond consumption controlled with the FT-IR technique based on changes in the surface areas of bands at ῡ = 895 cm^−1^ and ῡ = 2130 cm^−1^ characteristic for stretching vibrations of Si-H bond (usually 20–24 h).

## 3. Results and Discussion

This work aimed to examine the possibility of synthesizing a series of tri(alkenyl)substituted open-cage SQ derivatives varying in the length of the alkenyl chain and also in inert substituents (iBu and Ph) at silsesquioxane core. The type of inert and functional groups affects the synthetic procedure and also the physicochemical properties of these systems. As a result of this goal, a library of eight compounds was set and their thermal properties were also analyzed. It was meant to classify them as potential polymer modifiers and be chosen as those in terms of the polymer matrix thermal stability.

**Figure 1:**
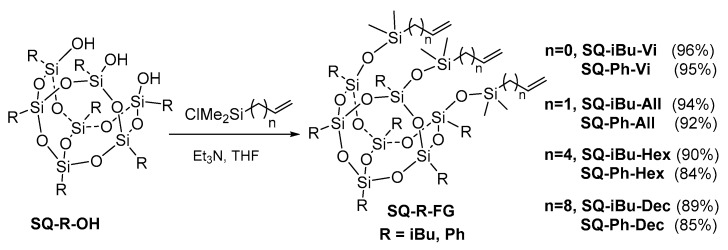
Synthesis of tri(alkenyl)functional silsesquioxanes **SQ-R-FG** via a hydrolytic condensation reaction.

A starting point was the design of the synthetic route for our aim. The hydrolytic condensation of trisilanol SQ’s with respective chlorosilane seemed to be the most probable way to achieve our aim. The first step of our work was to prepare substrates that are not commercially available, i.e., alkenylchlorosilanes with longer alkenyl chains, i.e., -hex-5-enyl and -dec-9-enyl. These compounds were efficiently obtained via hydrosilylation of a specific diene with chlorodimethylsilane while using Karstedt’s catalyst that we described earlier [48]. The next stage was the hydrolytic condensation reactions of heptaisobutyl- and heptaphenyltrisilanol (**SQ-R-OH**, R = iBu, Ph) with respective alkenylchloro(dimethyl)silane, as summarized in Figure 1.

All of these condensation reactions (Figure 1) where conducted using a concentrated THF solution of reagents. In the case of alkenyl(chloro)silane with longer alkenyl chain and heptaisobutyltrisilanol SQ, preliminary tests revealed that a reduction of the solvent amount to a minimum (just to dissolve the **SQ-iBu-OH**, estimated at 0.63 M) was crucial for obtaining fully substituted **SQ-iBu-FG** products. It was especially important for the **SQ-iBu-Hex** derivative (see Supplementary Materials, Figure S1b, in ^29^Si NMR spectrum the absence of −58 ppm resonance line derived from not reacted Si-OH moiety might be noted). The presence of not reacted Si-OH group in the SQ framework of **SQ-iBu-Hex** results in the difference in the chemical surrounding of other Si atoms and the appearance of an additional signal at 9.94 ppm and at ca. −68 ppm for T type Si atoms (see Supplementary Materials, Figure S1b). It is in accordance with the data of compounds described by Naka et al. [11]. Additionally, the important aspect of the synthetic procedure to ensure effective product formation was the proper cooling of the reaction mixture in an ice bath and the sequence of chlorosilane and amine addition. The reverse order of first chloro(alkenyl)silane and then dropping of amine, known in hydrolytic condensations methodology, allowed for obtaining of **SQ-R-FG** in the most efficient mean. Subsequently, the reaction was warmed up to RT and left for 24 h being stirred. The crude product was isolated from the reaction mixture by simple filtration from ammonium chloride and purified by precipitation in cold methanol. The basicity of amine was also verified and NEt_3_ was found to be more effective in comparison with (iPr)_2_NEt, which led to the inferior yield of **SQ-R-FG**. The stoichiometry of reagents was also tested and it was dependent on the length of the alkenyl group in the chlorosilane. The longer alkenyl chain was, the higher excess of reagents per 1 mol of **SQ-R-OH** were exploited (from 3.09, 3.15 equivalent for FG = Vi, 3.3, 3.9 for FG = All to 3.6, 4.5 equivalent for FG = Hex, Dec in chlorosilane and amine, respectively). All of the compounds obtained via the condensation reaction were isolated with high yields (84–96%). They are air-stable solids, waxy solids, or colorless oils, and can be synthesized on a multigram scale. They are soluble in organic solvents like DCM, CHCl_3_, THF, and toluene but not in, e.g., methanol, MeCN, and hexane (only for R = Ph). They were isolated and characterized by employing spectroscopic methods (^1^H, ^13^C, and ^29^Si NMR, as well as FT-IR, see Supplementary Materials). Figure 1 summarizes the information on their isolation yields.

**Figure 2:**
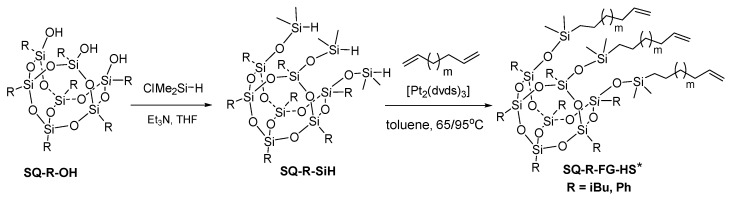
Synthesis of tri(alkenyl)functional silsesquioxanes via a sequence of hydrolytic condensation followed by a hydrosilylation reaction of dienes by **SQ-R-SiH**. *SQ-R-FG-HS accompanied by compounds of alkenyl chains with the isomerized C=C group. For respective structure, please see Supplementary Materials, Section 3a.

In the case of -hex-3-enyl (FG =Hex) and -dec-5-enyl (FG = Dec) functionalities, a parallel synthetic methodology that was based on the hydrosilylation reaction was also verified. For this purpose, we prepared silsesquioxane derivatives with three Si-H reactive groups (**SQ-R-SiH**, R = iBu, Ph), via hydrolytic condensation of trisilanol **SQ-iBu-OH** (R = iBu, Ph) form with ClSiMe_2_H basing on the procedure known from the literature [13] (see paragraph 2.3). The resulting **SQ-R-SiH** (R = iBu, Ph) were reagents for tests of hydrosilylation reaction with 1,5-hexadiene and 1,9-decadiene that was proceeded while using Karstedt’s catalyst (Figure 2).

**Figure 3:**
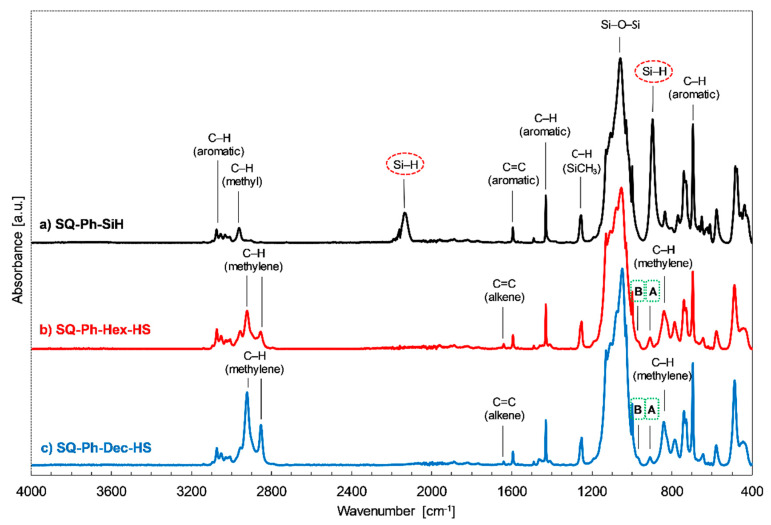
*Fourier Transform-Infrared* (FT-IR) spectra of **SQ-Ph-SiH**, **SQ-Ph-Hex-HS,** and **SQ-Ph-Dec-HS** after completion of hydrosilylation reaction.

In the experiments, the hydrosilylation reaction resulted in >99% conversion of Si-H bonds. The loading of [Pt_2_(dvds)_3_] was strictly controlled and estimated at 10−4 mol of per one Si-H group, along with the excess of diene, usually 3.6 equivalent The reaction was conducted at 65 °C in the case of 1,5-hexadiene and 95 °C for 1,9-decadiene until the total conversion of Si-H moiety. The reaction completion was monitored using FT-IR analysis (changes in the area of the bands at ῡ = 895 cm^−1^ and ῡ = 2130 cm^−1^, ascribed to the stretching vibrations of Si–H bond) and was usually performed for 20–24 h (exemplary spectra for the SQ-Ph derivatives in Figure 3).

The stacked spectra that are presented in Figure 3 exhibit band characteristics for **SQ-Ph-SiH** (marked on the spectra a). Additionally, there are new bands on the spectra, characteristics of -hex-3-enyl and -dec-5-enyl chains attached to the SQ core (**SQ-Ph-Hex-HS** and **SQ-Ph-Dec-HS**). These new bands may be attributed to stretching vibrations of C-H methylene groups in the aliphatic chain at ca. 2855 cm^−1^ and 2920 cm^−1^, C=C at 1640 cm^−1^ (weak intensity), and also arise of new bands resulting from rocking vibrations of C-H methylene groups at ca. 780 cm^−1^ and 840 cm^−1^. Additionally, one might note two bands denoted as **A** and **B** in Figure 3. Band A, at 920 cm^−1^, might be attributed to out-of-plane bending vibrations of the terminal = C-H moiety, and B at ca. 980 cm^−1^ is overlapped and probably resulting from out-of-plane bending vibrations of new (*trans*) = C-H group from diene isomerization that we also observed in the NMR spectra (see Figures 5 and 6).

**Figure 4:**
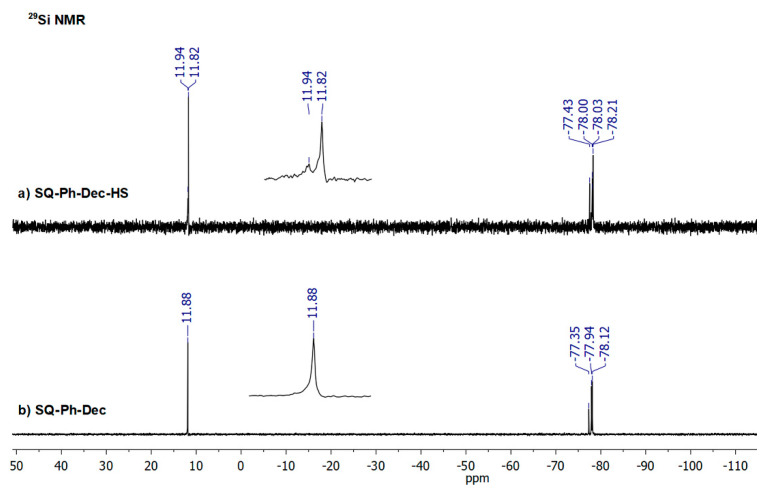
^29^Si NMR spectra of (**a**) **SQ-Ph-Dec-HS** obtained in a hydrosilylation and (**b**) **SQ-Ph-Dec** obtained via a condensation reaction.

As it was abovementioned, an isomerization of C=C in dienes and respective products of their hydrosilylation reaction was observed. It should be highlighted that the hydrosilylation process might not be selective and side reactions may occur, e.g., the isomerization of olefin, α- and/or β-addition products or dehydrogenative silylation products, etc. [49]. For the hydrosilylation of 1,5-hexadiene and 1,9-decadiene, despite >99% consumption of **SQ-R-SiH** reagent the crude products were accompanied by traces (for 1,9-decadiene) or notable, up to 30% amount (for 1,5-hexadiene) of olefin isomerization products (for possible structures, see Supplementary Materials, Section 3a). It was previously observed for the hydrosilylation of 1, 5-hexadiene by chlorosilane by Saiki et al. [50] and might be evaluated not only by FT-IR (Figure 3), but also by ^1^H, ^13^C, and ^29^Si NMR analysis. For **SQ-Ph-Dec-HS**, the evaluation of NMR data resulted in the identification of a by-product of double bond diene isomerization that was reflected in the additional resonance line at 11.94 ppm in ^29^Si NMR spectrum attributed to Si-Dec moiety (Figure 4). What should be underlined, this product was not obtained when **SQ-Ph-Dec** was obtained via hydrolytic condensation and the same was in the case of **SQ-Ph-Hex**.

**Figure 5:**
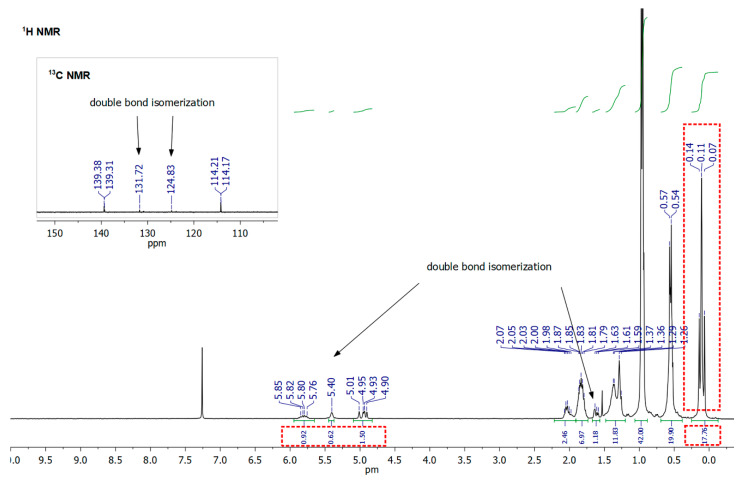
^1^H NMR spectrum of crude **SQ-iBu-Hex-HS** obtained via hydrosilylation of 1,5-hexadiene by **SQ-iBu-SiH** performed at higher concentrations, i.e., 0.22–0.25 M.

**Figure 6:**
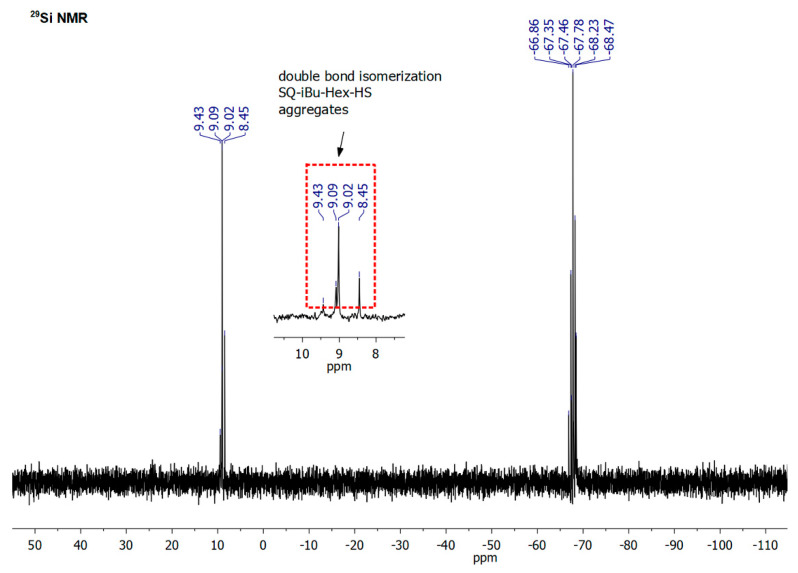
^29^Si NMR spectra of crude **SQ-iBu-Hex-HS** obtained via hydrosilylation of 1,5-hexadiene by **SQ-iBu-SiH** performed at higher concentration, i.e., 0.22–0.25 M.

Another aspect is that hydrosilylation of dienes should be also conducted in a proper dilution of **SQ-R-SiH** in toluene (*ca.* 0.04 M) to reduce possible hydrosilylation of one diene molecule by two separate particles of **SQ-R-SiH**. It was observed, especially in the case of 1,5-hexadiene (probable structure presented in Supplementary Materials, Section 3b). The formation of specific aggregates containing one or two SQs moieties may occur in a reaction performed in toluene at higher **SQ-R-SiH** concentration, i.e., 0.22–0.25 M. In the case of **SQ-R-Hex-HS** derivatives, when the reaction was conducted at the more concentrated system, despite >99,9% conversion of Si-H bonds, the crude product contained a notable amount of the by-product of 1,5-hexadiene isomerization, but also the aggregates if two or more molecules of SQ-R open cages. NMR spectra may confirm these assumptions (Figure 5 and Figure 6).

In the ^1^H NMR spectrum, there is a multiplication of signals at ca. 0.11ppm derived from methyl groups at Si (-O-SiMe_2_-Hex) due to the presence of saturated aliphatic hexane chains linking different SQs open cages, and, what is more important, the ratio of methyl protons (-O-SiMe_2_-Hex) to vinyl protons (-HC = CH_2_) in one molecule of **SQ-iBu-Hex** should be fixed at 2:1. In this case, the value ratio is underestimated, i.e., 8.8:1, which might indirectly suggest the decrease of vinyl moieties in the system, i.e., their consumption in the intermolecular hydrosilylation of 1,5-hexadiene by two distinct **SQ-iBu-SiH** molecules. This assumption might be confirmed by the GPC analysis (see. Supplementary Materials, Figures S3 and S4). As for the ^29^Si NMR spectrum of crude **SQ-iBu-Hex-HS**, there are also visible changes (Figure 6). Due to the presence of saturated aliphatic hexane chains linking different SQs open cages, there is a multiplication of signals at ca. 9.02 ppm derived from functional -O-Si-Hex, i.e., M-type Si atoms and there are also additional resonance lines originated from SQs core at ca. −68 ppm, i.e., *t*-type Si atoms.

The hydrolytic condensation and hydrosilylation reaction should not be considered as alternative procedures for obtaining tri(hex-3-enyl)- and tri(dec-5-enyl)- functional SQ’s compounds. Both of the methods require additional reaction step, i.e., for condensation, the synthesis of alkenylchlorosilanes with longer chains and hydrosilylation, and the synthesis of SQ-R-SiH (R = iBu, Ph). However, the formation of undesired by-products might not be circumvented in the case of hydrosilylation reaction and, as a result, the application of hydrolytic condensation should be the preferred methodology for the efficient synthesis of final products.

### TG and DSC Analysis

The discussed **SQ-R-FG** (R = iBu, Ph; FG = Hex, Dec) derivatives can be considered as i.e., valuable tripodal crosslinking agents for polymer systems processed via TM-catalyzed transformations (e.g., hydrosilylation) as well as UV or free radical initiated ones (e.g., thiolene addition), as mentioned above. The performance of the materials obtained in this manner is significantly affected by both the properties of the starting polymers and the crosslinkers used, among others, their thermal properties. As pointed by Naka, they are determined not only by the type of seven chemically inert organic substituents (isobutyl or phenyl) present in the structure of silsesquioxane, but also by the chemical structure of the three substituents at the SQ opening moieties [19]. In order evaluate the influence of hydrocarbon chain length of the alkenyl group anchored the Si-O-Si core of the discussed group of compounds on their thermal properties, they were subjected to the TG and DSC analyses.

**Figure 7:**
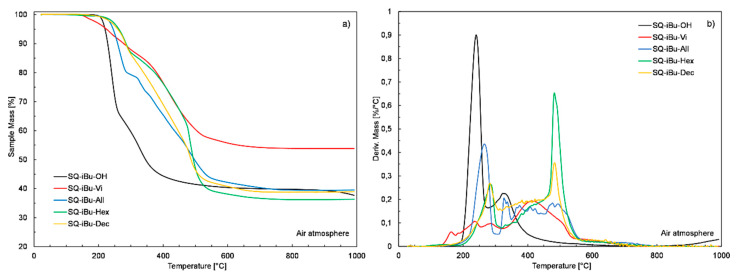
TG and DTG curves of **SQ-iBu-FG** silsesquioxane derivatives.

Figure 7 presents the TG and DTG curves acquired for SQ-iBu derivatives and Table 1 summarizes the results of their analysis. It can be observed that the length of the alkyl chain visibly influences the thermal stability of prepared compounds. The incorporation of three dimethylvinylsilyl groups into the structure of the **SQ-iBu-Vi** derivative resulted in a significant (by more than 70 °C) decrease in the decomposition onset temperature (T_onset_) as compared to the starting **SQ-iBu-OH**, while the presence of dimethylsilyl moieties of a longer alkyl chain (-allyl, -hex-3-enyl, and -dec-5-enyl ) in the **SQ-iBu-All**, **SQ-iBu-Hex**, and **SQ-iBu-Dec** derivatives caused an increase in their T_onset_ values to 236, 250, and 246 °C, respectively.

Based on the course of TG and DTG curves, it can be also observed that the decomposition process of the dimethylalkenylsilyl functionalized silsesquioxanes is much more complex (multimodal), significantly spread in time, and shifted to the higher temperatures as compared to **SQ-iBu-OH** derivative. The char yield values at 1000 °C measured for starting **SQ-iBu-OH** and functionalized **SQ-iBu-All**, **SQ-iBu-Hex**, and **SQ-iBu-Dec** samples were very similar and ranged from 36% to 40%, regardless of the alkenyl chain length. Interestingly, the char yield value that was measured at 1000 °C for the **SQ-iBu-Vi** sample was over 14% higher and reached 54%. It should be noted that the thermal behavior of the **SQ-iBu-Vi** sample is quite different from the rest of the SQ-iBu derivatives. The decrease in T_onset_ temperature and increase in char yield value observed for **SQ-iBu-Vi** derivative can be explained by the relatively higher reactivity and lower thermal stability of dimethylvinylsilyl groups. They may undergo either free-radical polymerization or thermal cleavage, followed by hydrolytic polycondensation, leading to the formation of more thermally stable, crosslinked products, and finally a higher amount of char at elevated temperatures.

**Figure 8:**
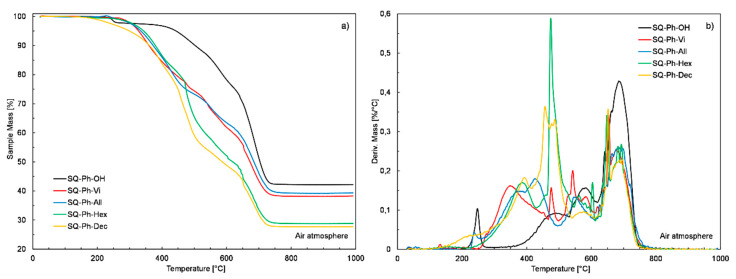
TG and DTG curves of **SQ-Ph-FG** silsesquioxane derivatives.

Opposite to the **SQ-iBu-FG** derivatives discussed above, the incorporation of the dimethylalkenylsilyl moieties into the heptaphenyltrisilanol silsesquioxane, regardless of the alkenyl chain length, resulted in the formation of derivatives of lower thermal stability when compared to the starting material (**SQ-Ph-OH**). Their T_onset_ temperatures ranged from 300 to 327 °C, while the T_onset_ temperature of the main decomposition step measured for **SQ-Ph-OH** derivative was at least 100 °C higher, as shown in Figure 8 (see Table 1). The decomposition process of the SQ-Ph functionalized silsesquioxanes also became more complex, spread in time and was shifted to the lower temperatures when compared to **SQ-Ph-OH** derivative. The values of the char yield at 1000 °C obtained for all functionalized **SQ-Ph-Vi**, **SQ-Ph-All**, **SQ-Ph-Hex**, and **SQ-Ph-Dec** silsesquioxanes (38, 39, 29, and 28%, respectively) were lower than those that were measured for starting **SQ-Ph-OH** derivative (43%) unlike in the case of **SQ-iBu-OH** derivative. Moreover, it can be observed that char yields of the **SQ-Ph-Vi** and **SQ-Ph-All** samples were notably higher than those of **SQ-Ph-Hex** and **SQ-Ph-Dec** ones, which result from the lower carbon content in their structure. The decrease in T_onset_ temperatures and char yields observed for all functionalized **SQ-Ph**-**FG** derivatives when compared to the **SQ-Ph-OH** could be explained by the occurrence of its thermally initiated dehydration, followed by the hydrolytic polycondensation leading to the formation of the higher amount of thermally stable char. The presence of the characteristic step transition observed in the course of the **SQ-Ph-OH** TG curve at 250 °C could be attributed to the mentioned dehydration process and justify the above hypothesis.

Based on the results of thermogravimetric analysis, it seems that the length of alkenyl chains only marginally affects the thermal stability of the tested group of compounds and the type of the remaining seven functional groups (iBu or Ph) or rather the superimpose of both parameters has a profound impact on silsesquioxanes thermal stability.

**Figure 9:**
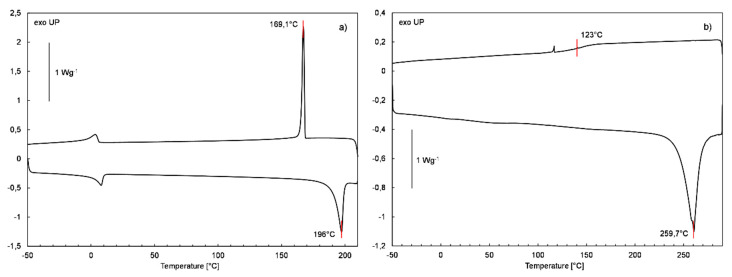
DSC curves of the heating and cooling run of (**a**) **SQ-iBu-OH** and (**b**) **SQ-Ph-OH** derivative.

In order to further explain the above-discussed relationships and evaluate the impact of the alkenyl chain length on the melting and crystallization characteristics of both series of compounds, they were subjected to DSC analysis. First, the starting **SQ-iBu-OH** and **SQ-Ph-OH** derivatives were analyzed and Figure 9 presents their DSC thermograms. It can be seen that **SQ-iBu-OH** derivative melts at 196 °C and crystalize during cooling at 169 °C. In the case of **SQ-Ph-OH** derivative, an endothermic signal with the maximum at 260 °C on a heating curve and a characteristic step transition at 123 °C (midpoint) on a cooling curve can be observed. This means that the **SQ-Ph-OH** derivative melts at 260 °C with the decomposition and formation of an amorphous polymeric phase of glass transition at 123 °C, which is consistent with the results of TG analysis that were obtained for this compound and suggested the explanation of its thermal stability. It should be noted that melting and crystallization temperatures measured for **SQ-iBu-OH** derivatives are quite different from those provided by suppliers in the product technical documentation (SDS). A possible explanation of this difference is that used for the syntheses and subjected to the TG and DSC analysis derivative was recrystallized before use, as described earlier.

**Figure 10:**
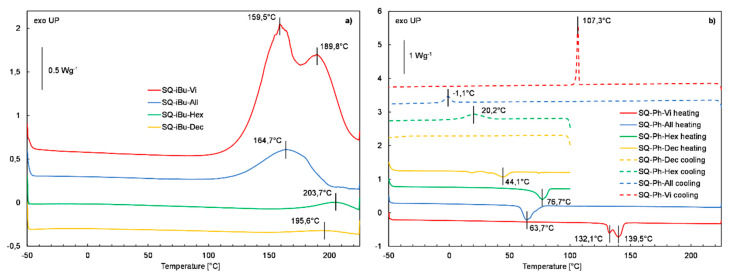
DSC curves of (**a**) the first heating run of **SQ-iBu-FG** derivatives, and (**b**) the second heating and cooling run of **SQ-Ph-FG** derivatives.

Subsequently, a series of functionalized derivatives **SQ-iBu-FG** and **SQ-Ph-FG** were subjected to DSC analysis, and their thermograms are presented in Figure 10a,b respectively. Table 2 summarizes all of the DSC analysis results.

DSC analysis of the **SQ-iBu-FG** derivatives series revealed the presence of only exothermic signals on the heating threads of DSC curves of all the measured samples in the range from 100 to 200 °C and no signals were observed on their cooling threads (Figure 10a). This indicates that the tested derivatives are not crystalline and undergo exothermic chemical reactions in the mentioned range of temperatures, leading to the formation of amorphous products. Moreover, the observed gradual decrease in the enthalpy (ΔH_r_) of reaction from 456 to 115, 15, and 9 Jg−1 for **SQ-iBu-Vi**, **SQ-iBu-All**, **SQ-iBu-Hex**, and **SQ-iBu-Dec**, respectively, indicates that the reactivity of alkenyl groups is inversely proportional to their hydrocarbon chain length. The decrease in the ΔH_r_ values stays in good agreement with the char yields of **SQ-iBu-FG** derivatives established based on TG analysis results (see Table 1). Nevertheless, to additionally confirm the occurrence of a chemical process involving the reactivity of unsaturated CH = CH_2_ groups of the **SQ-iBu-Vi** sample, the one of potentially highest reactivity, it was heated in an air atmosphere at 200 °C for 30 min. and subsequently subjected to ^1^H NMR analysis to evaluate changes in its structure. The results of this analysis presented in Figure S2 (see Supplementary Materials) revealed the complete disappearance of signals of chemical shift in the range from 5.7 to 6.2 ppm characteristic for vinyl protons and the formation of new signals at 1.25, 1.33, and 2.18 ppm as the result of the formation of new methylene (CH_2_) units. This might confirm the hypothesis of the occurrence of thermally initiated vinyl group polymerization. However, it should also be noted that the intensity of the signal present in the ^1^H NMR at 0.2 ppm attributed to the protons present in dimethylsilyl groups was also decreased. This means that part of the dimethylsilyl groups might have been thermally cleaved and formed active sites on silsesquioxane core that could subsequently undergo polycondensation process typical for organosilicon derivatives, also leading to the formation of high molecular, random structures.

In contrast to the **SQ-iBu-FG** derivatives, the incorporation of three dimethylalkenylsilyl groups into the structure of heptaphenylsilsequioxane resulted in the formation of crystalline products and no thermal degradation process was observed below their melting points, as the characteristic exothermic crystallization signals were observed on the cooling threads of their DSC thermograms (Figure 10b) for all of the measured samples, except the **SQ-Ph-Dec** one. In this case, the crystallization temperature was observed on its cooling DSC curve in the measured temperature range. Nevertheless, the curve inflection at the end of the temperature program might indicate the occurrence of a glass transition. It can be also observed that melting and crystallization temperatures of **SQ-Ph-All**, **SQ-Ph-Hex**, and **SQ-Ph-Dec** silsesquioxanes of longer alkenyl chains were significantly lower as compared to the **SQ-Ph-Vi** derivative. The incorporation of dimethylsilyl groups into the **SQ-Ph-OH** structure effectively inhibited its hydrolytic polycondensation at elevated temperatures what explain the decrease in T_onset_ temperatures of functionalized **SQ-Ph-FG** derivatives (see Figure 8 and Table 1).

## 4. Conclusions

In summary, we designed and synthesized novel tri(alkenyl)functional silsesquioxanes **SQ-R-FG** (R = iBu, Ph; FG = Vi, All, Hex, Dec), with various chain lengths and with two types of inert groups. We revealed simple and efficient routes of their synthesis and isolation with high purity using a hydrolytic condensation reaction. Additionally, a parallel synthetic procedure was tested for the synthesis of tri(hex-3-enyl)- and tri(dec-5-enyl )functional SQs, based on the hydrosilylation of 1,5-hexadiene and 1,9-decadiene with **SQ-R-SiH** with three reactive Si–H moieties. However, this procedure appeared to be of lower selectivity of desired products due to possible side reactions and it should not be applied for obtaining the unsaturated SQ-based open-cage compounds.

All of the obtained compounds were analyzed with TG and DSC techniques in terms of the evaluation of the influence of the alkenyl chain length in the dimethylsilyl groups on their thermal stability, melting and crystallization temperatures. The obtained results of the thermal analysis revealed that the length of alkenyl chains do affect the thermal stability as well as the melting and crystallization temperatures of the tested group of compounds. Additionally, it was proved that the type of the remaining seven functional groups (iBu or Ph) has a crucial impact on the considered parameters. It was also found that the trisubstituted derivatives of SQ-iBu can undergo thermally initiated polymerization and polycondensation processes with the formation of homogeneous, transparent polymeric materials. These observations will be the starting point for further research while using the discussed group of compounds as substrates for protective coating materials synthesis and assessing the impact of the structure of reactive groups introduced on their physicochemical properties.

## Figures and Tables

**Table 1:** Results of TG and DTG analysis of **SQ-iBu-FG** and **SQ-Ph-FG** silsesquioxane derivatives.

Sample	Mass Loss Temperature [°C]			T_onset_ [°C]	CHAR Yield [%] ^a^
	1%	5%	10%		
SQ-iBu-OH	206.6	220	240.4	218.2	37.7
SQ-iBu-Vi	165.9	223.2	392	147.5	54.2
SQ-iBu-All	218.1	245.2	288.2	236.4	39.5
SQ-iBu-Hex	220	262.8	374	250.2	36.3
SQ-iBu-Dec	210.8	259	339.2	245.9	38.9
SQ-Ph-OH	223.4	451.8	590.2	426.3	42.5
SQ-Ph-Vi	281.4	327.9	442.6	300.9	38.3
SQ-Ph-All	267.7	334.9	435.7	307.2	39.3
SQ-Ph-Hex	267.6	343.5	454.1	327.2	28.8
SQ-Ph-Dec	198.6	299.9	424.1	305.8	27.7

^a^ @1000 °C.

**Table 2:** Results of DSC analysis of **SQ-iBu-FG** and **SQ-Ph-FGs** silsesquioxane derivatives.

Sample	Melting		Reaction		Crystallization		Glass Transition [°C]
	Temp. [°C]	ΔH [Jg−1]	Temp. [°C]	ΔH [Jg−1]	Temp. [°C]	ΔH [Jg−1]	
SQ-iBu-OH	196	−32.3	-	-	169.1	26	-
SQ-iBu-Vi	-	-	159.5 189.8	275.9 180.3	-	-	-
SQ-iBu-All	-	-	164.7	114.9	-	-	-
SQ-iBu-Hex	-	-	203.7	15.1	-	-	-
SQ-iBu-Dec	-	-	195.6	8.8	-	-	-
SQ-Ph-OH	259.7	−64.8	-	-	-	-	123
SQ-Ph-Vi	132.1 139.5	−8.2 −17.1	-	-	107.3	18	-
SQ-Ph-All	63.7	−26.8	-	-	−1.1	6.2	-
SQ-Ph-Hex	76.7	−22.2	-	-	20.2	15.5	-
SQ-Ph-Dec	18.6 44.1 73.3	−1.3 −9.8 −0.4	-	-	-	-	- *

* Possible glass transition below −40 °C.

## Supplementary Materials

Detailed analytical data with NMR spectra of all isolated products along with the additional NMR spectra and GPC chromatograms are available online at https://www.mdpi.com/2073-4360/12/5/1063/s1.
