# Supplementary material for: Preparation of Tri(alkenyl)functional Open-Cage Silsesquioxanes as Specific Polymer Modifiers
Source: Polymers (Basel). 2020 May 6;12(5):1063. doi: 10.3390/polym12051063 (PMC7285154; doi:10.3390/polym12051063)
Supplement: Supplementary file 1 [file polymers-12-01063-s001.pdf]

Supporting Information for

Preparation of tri(alkenyl)functional open-cage silsesquioxanes as specific polymer modifiers

Katarzyna Mituła[1,2], Michał Dutkiewicz[2,3], Julia Duszczak[1,2], Monika Rzonsowska[1,2], Beata Dudziec [1,2,*]

[1]Faculty of Chemistry, Adam Mickiewicz University in Poznan, Uniwersytetu Poznańskiego 8, 61-614 Poznan, Poland
[2]Centre for Advanced Technologies, Adam Mickiewicz University in Poznan, Uniwersytetu Poznańskiego 10, 61-614 Poznan, Poland
[3]Adam Mickiewicz University Foundation, Rubiez 46, 61-612 Poznan, Poland

*Correspondence: beata.dudziec@gmail.com; Tel.: +48 618231878

Table of Contents:

1. Table of obtained products S 2

   1.1 Spectroscopic analysis of obtained products along with the copies of ^{1}H, ^{13}C, ^{29}Si NMR spectra S 3

2. Additional NMR spectra S 23

   2.1 Product **SQ-iBu-Hex** obtained in a different conditions of condensation reaction S 23

   2.2 Thermal cross-condensation of **SQ-iBu-Vi** S 24

3. Products of hydrosilylation of dienes by **SQ-R-SiH** conducted in different conditions S 25

4. GPC chromatograms S 26

5. References S 27

1. Table of obtained products

Prod. Abbreviation	Structure	Page
**SQ-iBu-SiH**	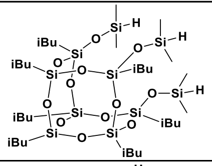	S 3
**SQ-Ph-SiH**	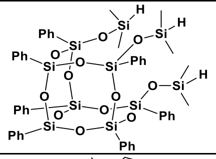	S 5
**SQ-iBu-Vi**	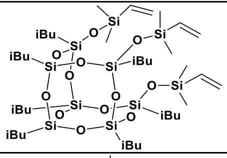	S 7
**SQ-Ph-Vi**	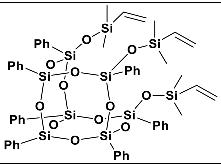	S 9
**SQ-iBu-All**	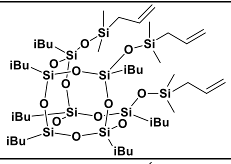	S 11
**SQ-Ph-All**	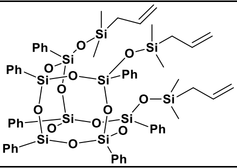	S 13
**SQ-iBu-Hex**	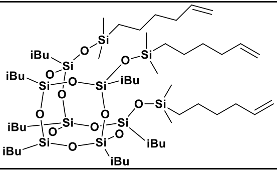	S 15
**SQ-Ph-Hex**	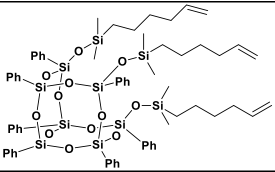	S 17
**SQ-iBu-Dec**	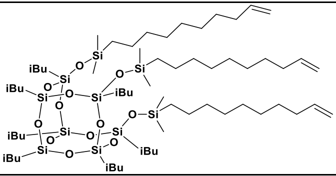	S 19
**SQ-Ph-Dec**	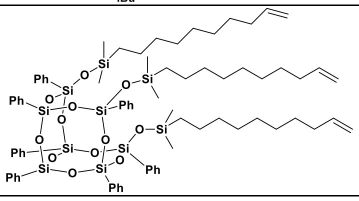	S 21

1.1 Spectroscopic analysis of obtained products along with the copies of ^{1}H, ^{13}C, ^{29}Si NMR spectra

SQ-iBu-SiH

3,7,14-tris[(hydro)dimethylsiloxy]-1,3,5,7,9,11,14-hepta(isobutyl)tricyclo[7.3.3^{15,11}]heptasiloxane

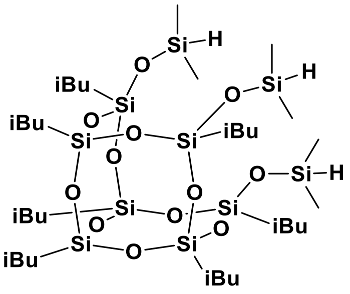

Colorless waxy solid, isolated yield **78%.**
^{1}**H NMR** (300.2 MHz, $CDCl_3$, δ, ppm): 0.23 (s, 18H, -SiC***H₃***), 0.55-0.58 (m, 14H, -C$\boldsymbol{H_2}$- (iBu)), 0.96-0.98 (m, 42H, -C$\boldsymbol{H_3}$ (iBu)), 1.80-1.89 (m, 7H, -C***H***- (iBu)), 4.74-4.75 (m, 3H, Si-***H***). ^{13}**C NMR** (100.6 MHz, $CDCl_3$, δ, ppm): -0.79 (-Si**C**H_3), 22.59, 23.74, 24.03-24.21, 24.76, 25.77, 25.99-26.11 (iBu) ^{29}**Si NMR** (79.5 MHz, $CDCl_3$, δ, ppm): -5.48 (**Si**-H), -67.12, -67.68, -68.01. **FT-IR** (cm^{-1}): 2953.62, 2902.15, 2869.31 (-C-H), 2138.98 (Si-H), 1465.75 (-C-H), 1252.58, 1227.70 (Si-C), 1050.99 (Si-O). The assignments are consistent with literature[1].

^{1}H NMR

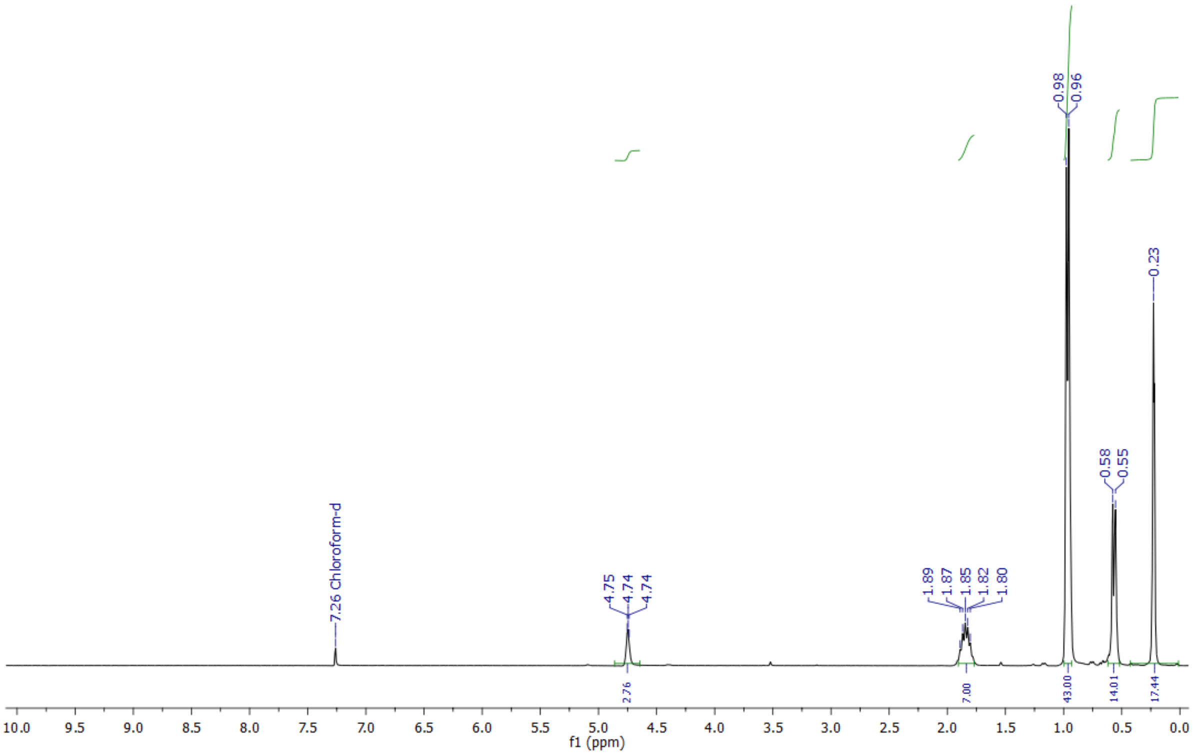

^{13}C NMR

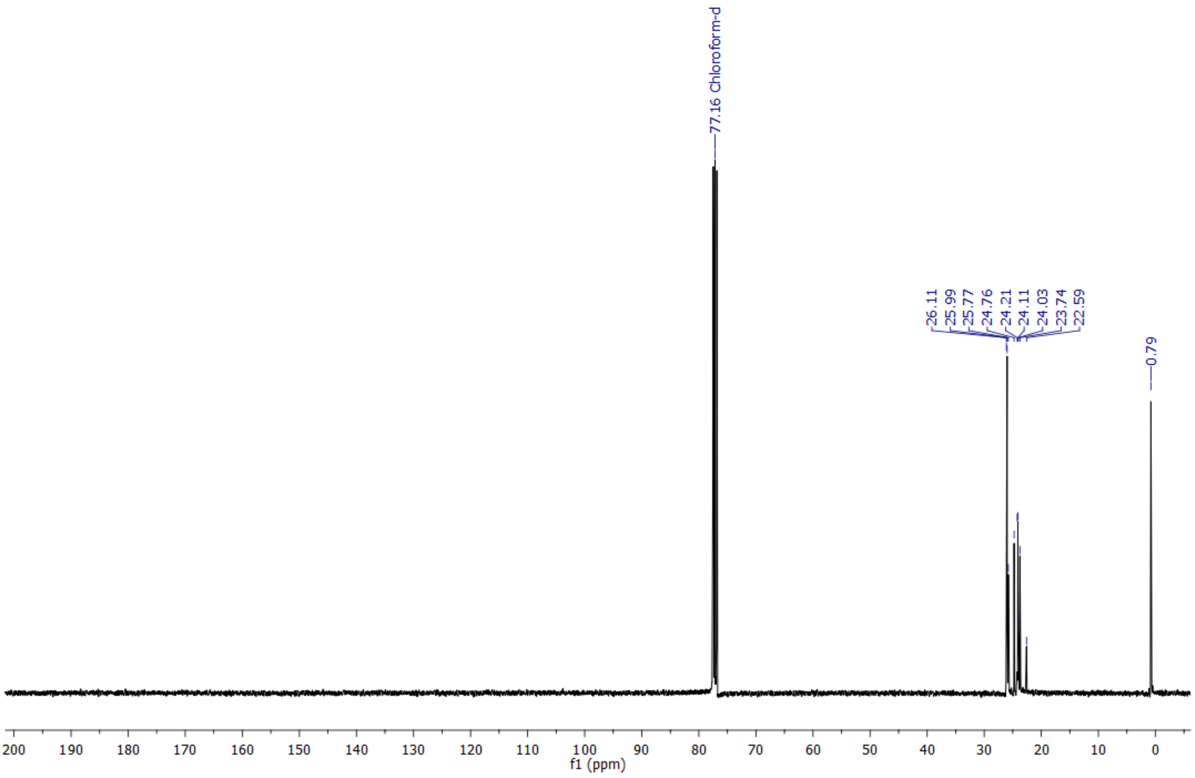

^{29}Si NMR

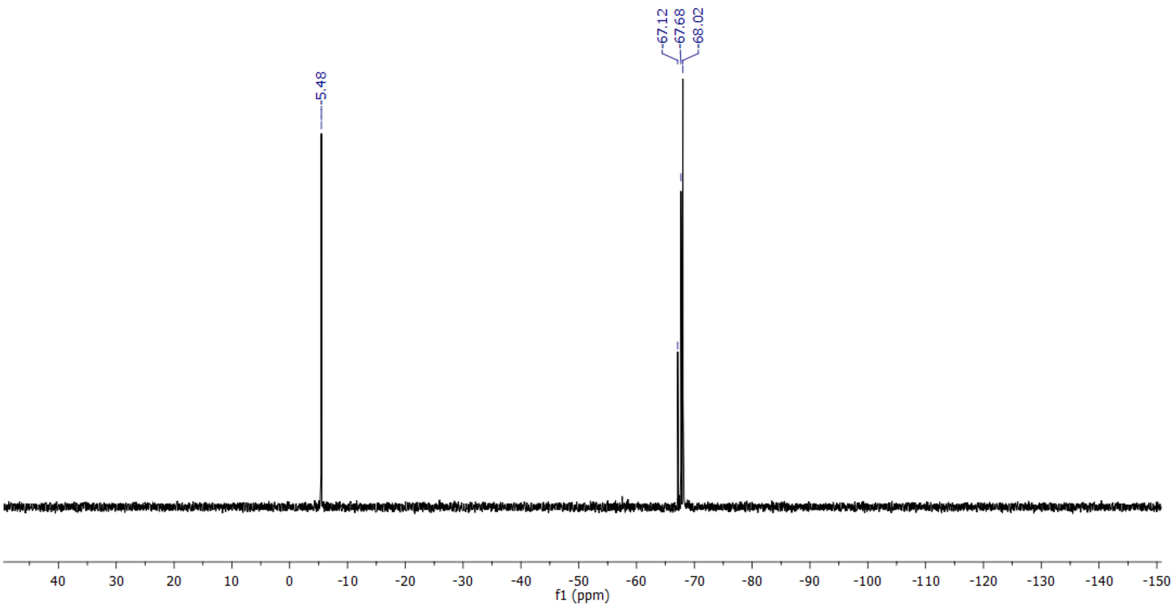

SQ-Ph-SiH

3,7,14-tris[(hydro)dimethylsiloxy]-1,3,5,7,9,11,14-hepta(phenyl)tricyclo[7.3.3^{15,11}]heptasiloxane

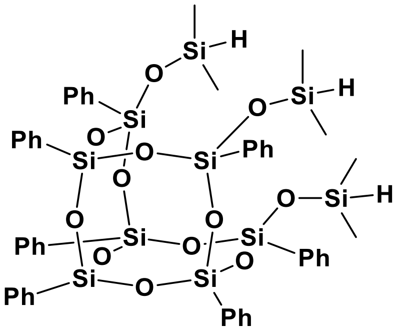

White solid, isolated yield **94%.**
^{1}H NMR (300.2 MHz, $CDCl_3$, δ, ppm): 0.35-0.36 (m, 18H, -SiC***H₃***), 4.93-4.95 (m, 3H, Si-***H***), 7.13-7.61 (m, 35H, Ph). **^{13}C NMR** (100.6 MHz, $CDCl_3$, δ, ppm): 0.82 (-Si**C**H_3), 127.66-128.02, 130.23-130.33, 131.10, 132.59, 134.05-134.16 (Ph). **^{29}Si NMR** (79.5 MHz, $CDCl_3$, δ, ppm): -2.83 (**Si**-H), -77.27, -77.61, -78.24.
FT-IR (cm^{-1}): 3074.58, 3054.01 (C-H phenyl), 2961.89 (-C-H), 2135.55 (Si-H), 1594.72 (C=C phenyl), 1490.37 (-C-H), 1430.27 (C=C phenyl), 1253.96 (Si-C), 1057.80, 1029.00 (Si-O), 998.45 (C-H phenyl). The assignments are consistent with the literature[2].

^{1}H NMR

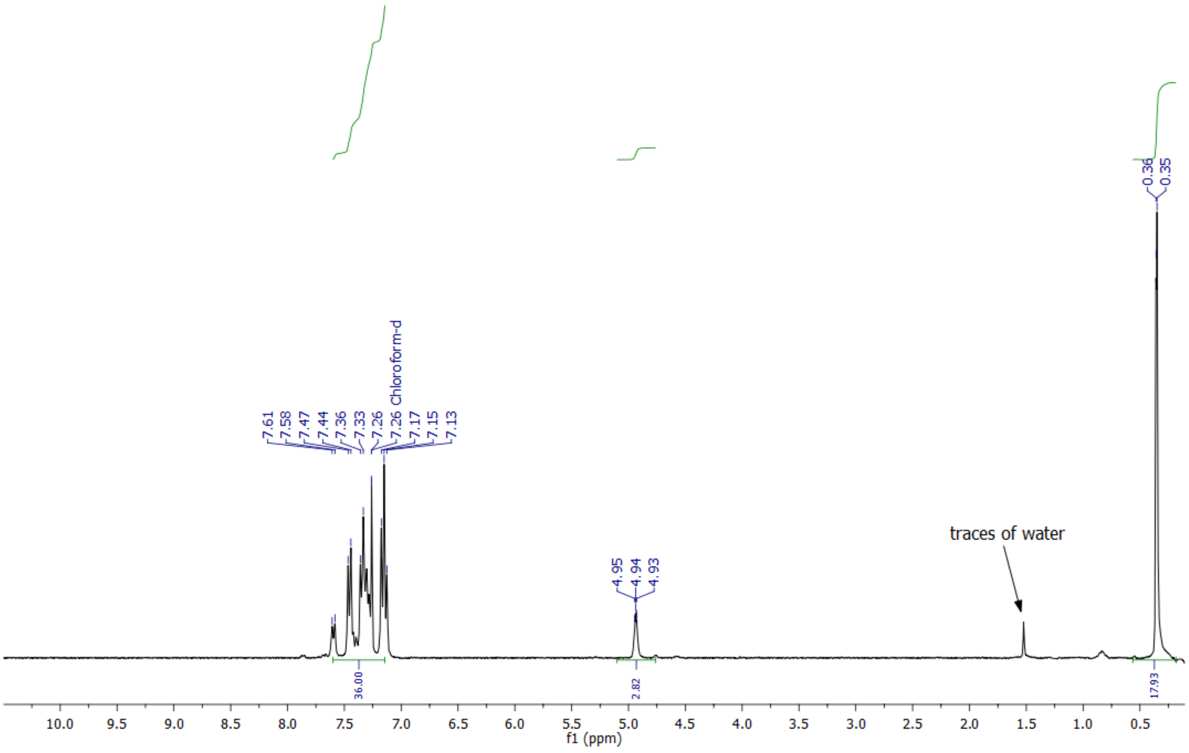

^{13}C NMR

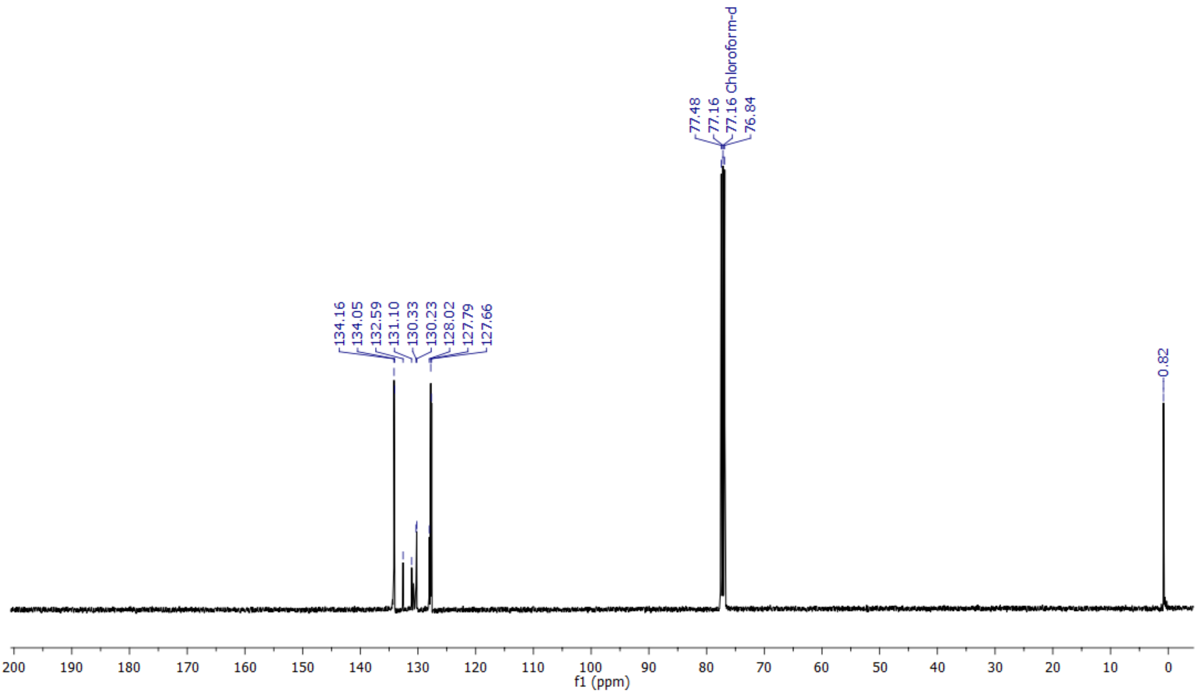

^{29}Si NMR

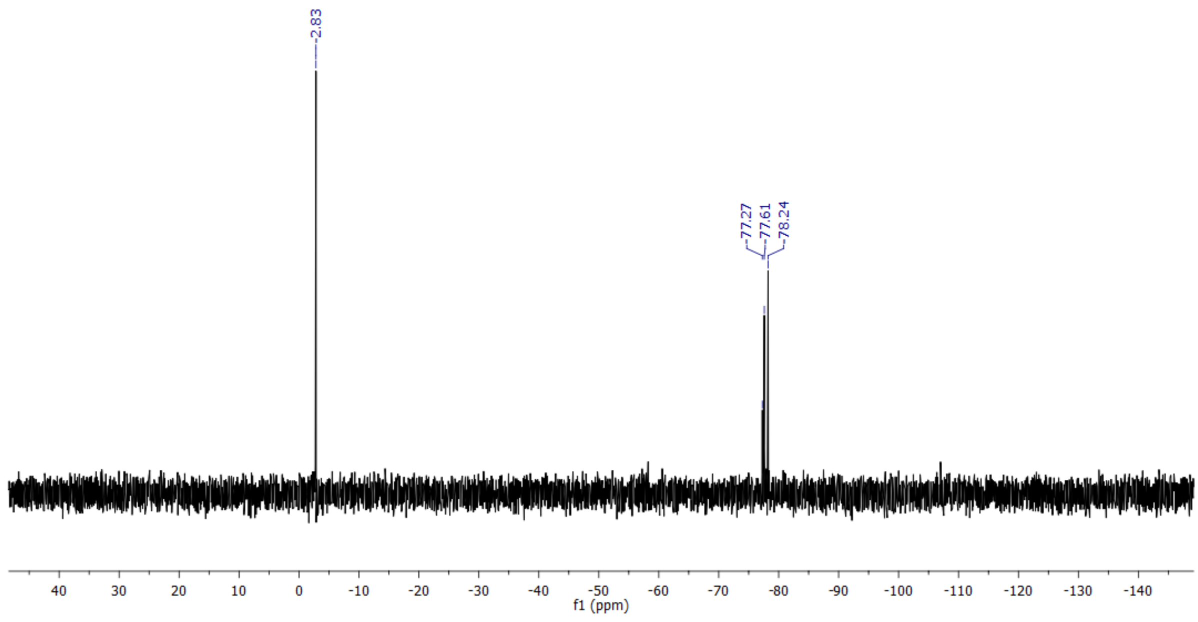

SQ-iBu-Vi

3,7,14-tris[dimethyl(vinyl)siloxy]-1,3,5,7,9,11,14-hepta(isobutyl)tricyclo[7.3.3^{15,11}]heptasiloxane

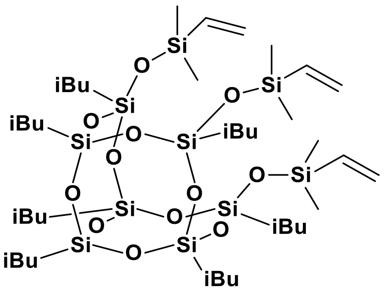

Colorless waxy solid, isolated yield **96%**

^{1}H NMR (300.2 MHz, $CDCl_3$, δ, ppm): 0.19 (s, 18H, -SiC***H*$_3$**), 0.53-0.57 (m, 14H, -C***H*$_2$**- (iBu)), 0.95-0.97 (m, 42H, -C***H*$_3$** (iBu)), 1.80-1.86 (m, 7H, -C***H*-** (iBu)), 5.74 (dd, $J_{H,H}$ = 20.2 Hz, 4.1 Hz, 3H, H_2C=C***H***), 5.92 (dd, $J_{H,H}$ = 14.9 Hz, 4.1 Hz, 3H, ***H*$_2$**C=CH-), 6.14 (dd, $J_{H,H}$ = 20.2 Hz, 14.9 Hz, 3H, ***H*$_2$**C=CH-). **^{13}C NMR** (100.6 MHz, $CDCl_3$, δ, ppm): -0.53 (-Si**C**H$_3$), 22.62, 23.89, 24.10-24.21, 25.13, 25.79, 26.02-26.18 (iBu), 131.90 (H_2**C**=CH-), 139.30 (H_2C=**C**H-). **^{29}Si NMR** (79.5 MHz, $CDCl_3$, δ, ppm): -2.68 (-O***Si***$(CH_3)_2$-), -67.29, -67.81, -68.04.

FT-IR (cm^{-1}): 3050.94 (=C-H), 2953.09, 2868.69 (-C-H), 1595.49 (C=C), 1465.73 (-C-H), 1252.59 (Si-C), 1045.58 (Si-O).

The assignments are consistent with those in the literature[2,3].

Chemical formula: $C_{40}H_{90}O_{12}Si_{10}$. **MALDI-ToF MS:** Calcd. for $C_{40}H_{90}NaO_{12}Si_{10}$: *m/z* 1065.4023, found: 1065.4026.

^{1}H NMR

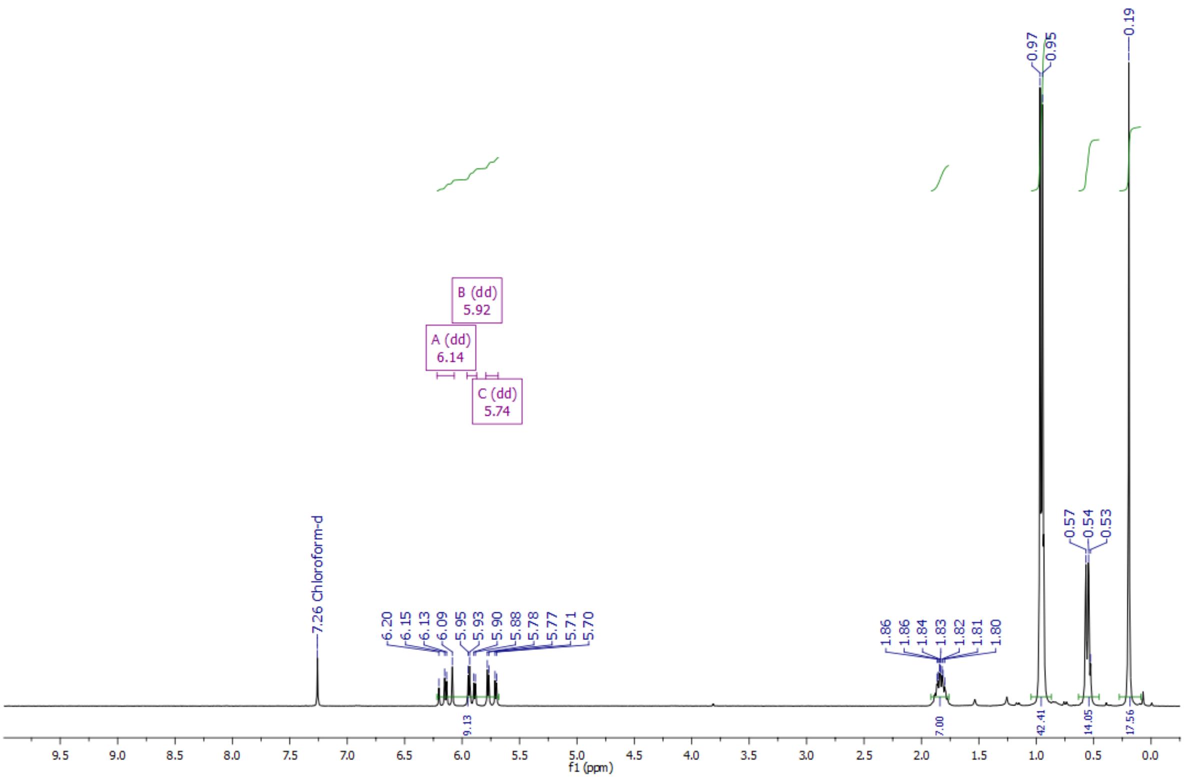

^{13}C NMR

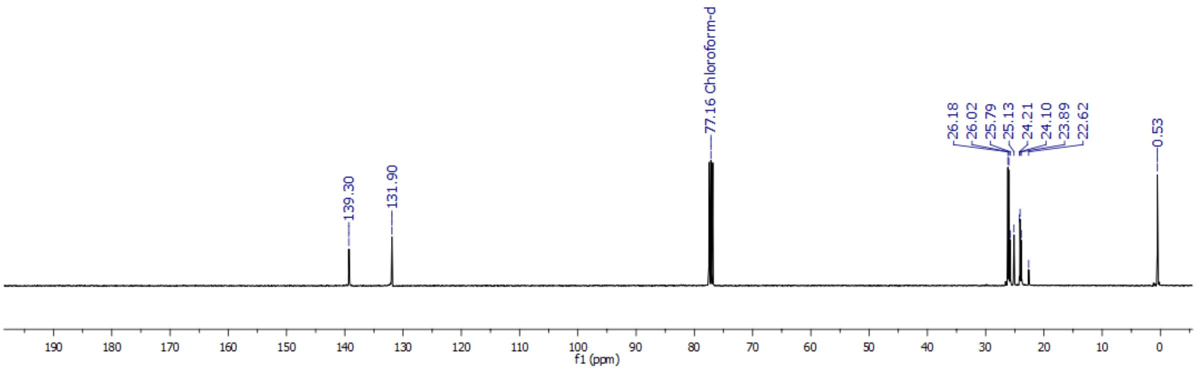

^{29}Si NMR

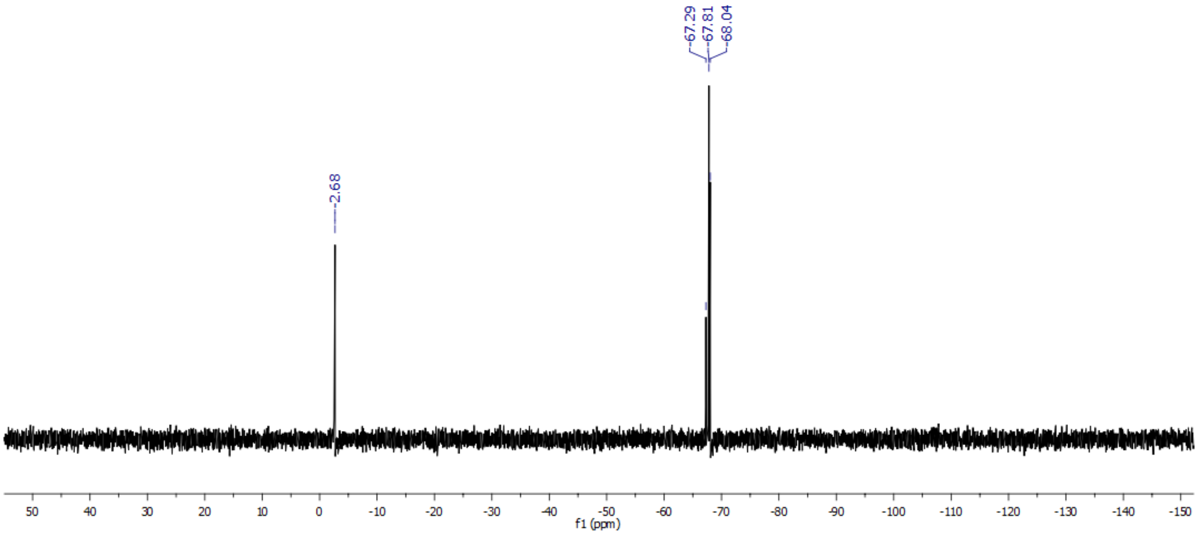

SQ-Ph-Vi

3,7,14-tris[dimethyl(vinyl)siloxy]-1,3,5,7,9,11,14-hepta(phenyl)tricyclo[7.3.3^{15,11}]heptasiloxane

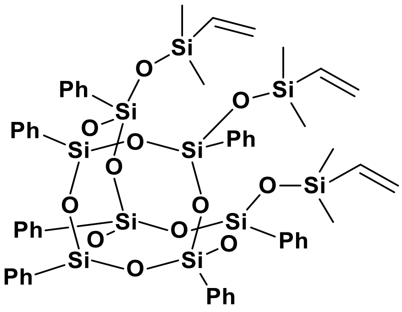

White solid, isolated yield **95%**

^{1}H NMR (300.2 MHz, $CDCl_3$, δ, ppm): 0.31 (s, 18H, -SiC$\boldsymbol{H_3}$), 5.83 (dd, $J_{H,H}$ = 20.2 Hz, 4.0 Hz, 3H, H_2C=C***H***-), 5.97 (dd, $J_{H,H}$ = 14.9 Hz, 4.0 Hz, 3H, $\boldsymbol{H_2}$C=CH-), 6.21 (dd, $J_{H,H}$ = 20.2 Hz, 14.9 Hz, 3H, $\boldsymbol{H_2}$C=CH-), 7.08-7.58 (m, 35H, **Ph**). **^{13}C NMR** (100.6 MHz, $CDCl_3$, δ, ppm): 0.54 (-Si**C**H_3), 127.60-128.67, 128.01, 130.08-130.23, 131.27 (Ph), 132.55 (H_2**C**=CH-), 132.88, 134.13-134.17 (Ph), 138.75 (H_2C=**C**H-) . **^{29}Si NMR** (79.5 MHz, $CDCl_3$, δ, ppm): -0.17 (-O***Si***$(CH_3)_2$-), -77.41, -77.80, -78.16.

FT-IR (cm^{-1}): 3073.49, 3051.43 (C-H phenyl), 2960.76, 2853.27 (-C-H), 1593.97 (C=C phenyl), 1489.99 (-C-H), 1430.09 (C=C phenyl), 1264.25 (Si-C), 1129.68, 1049.14 (Si-O), 997.91 (C-H phenyl).

The assignments are consistent with those in the literature[2].

Chemical formula: $C_{54}H_{62}O_{12}Si_{10}$. **MALDI-ToF MS:** Calcd. for $C_{54}H_{62}HNa^{+}O_{12}Si_{10}$: *m/z* 1206.1904, found: 1206.1906.

^{1}H NMR

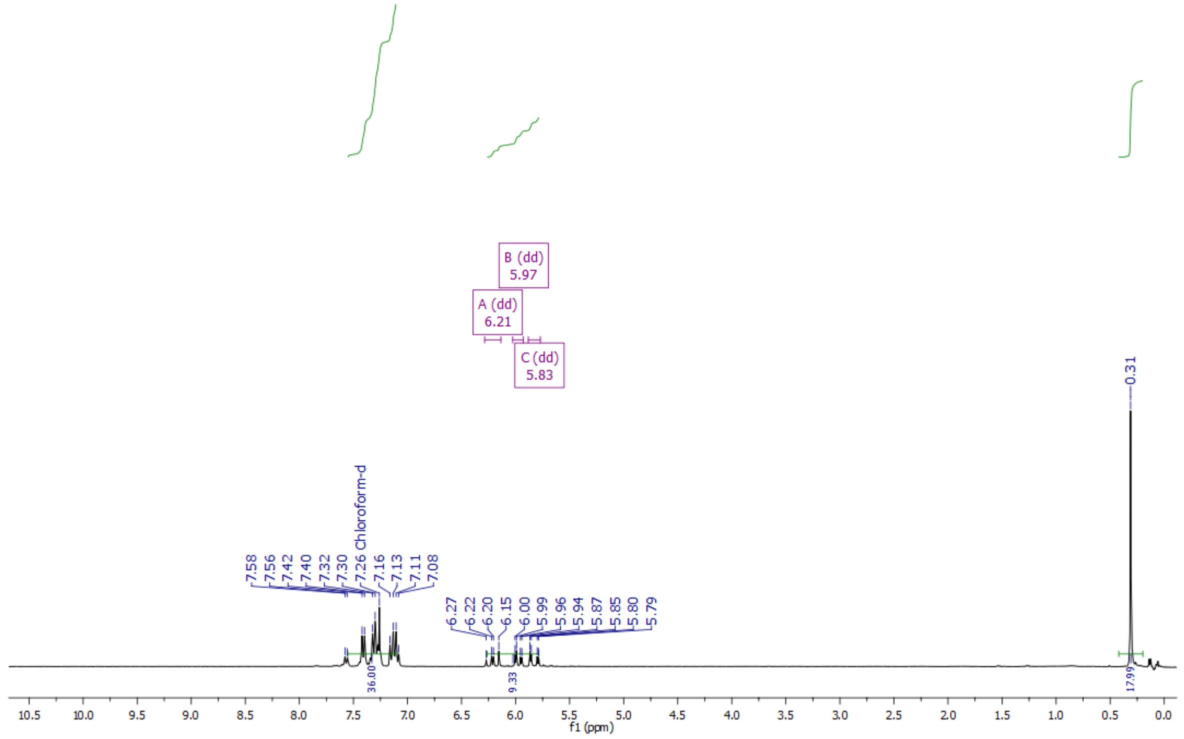

^{13}C NMR

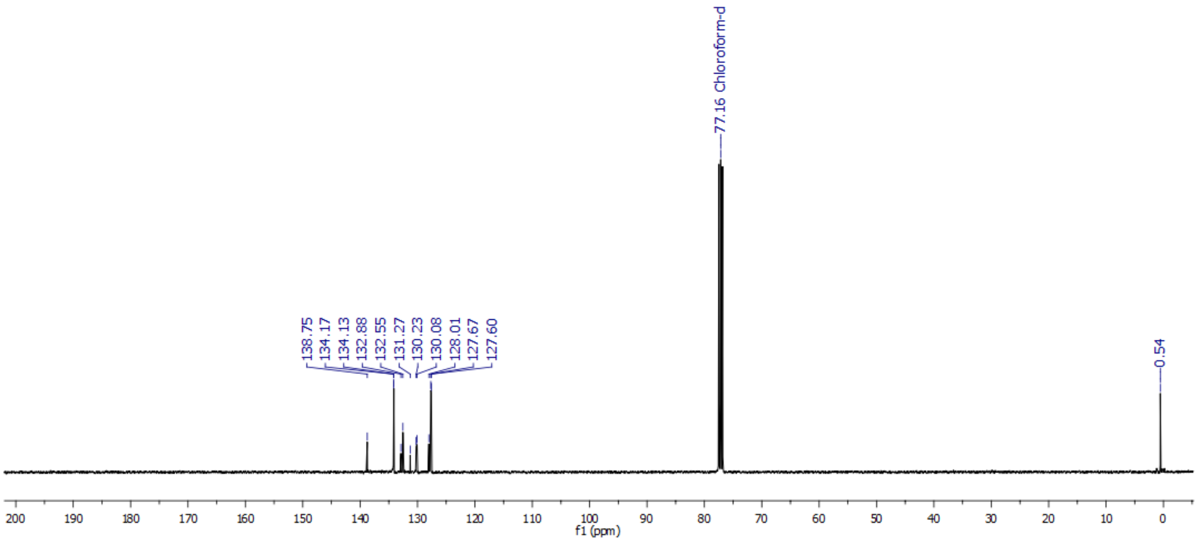

^{29}Si NMR

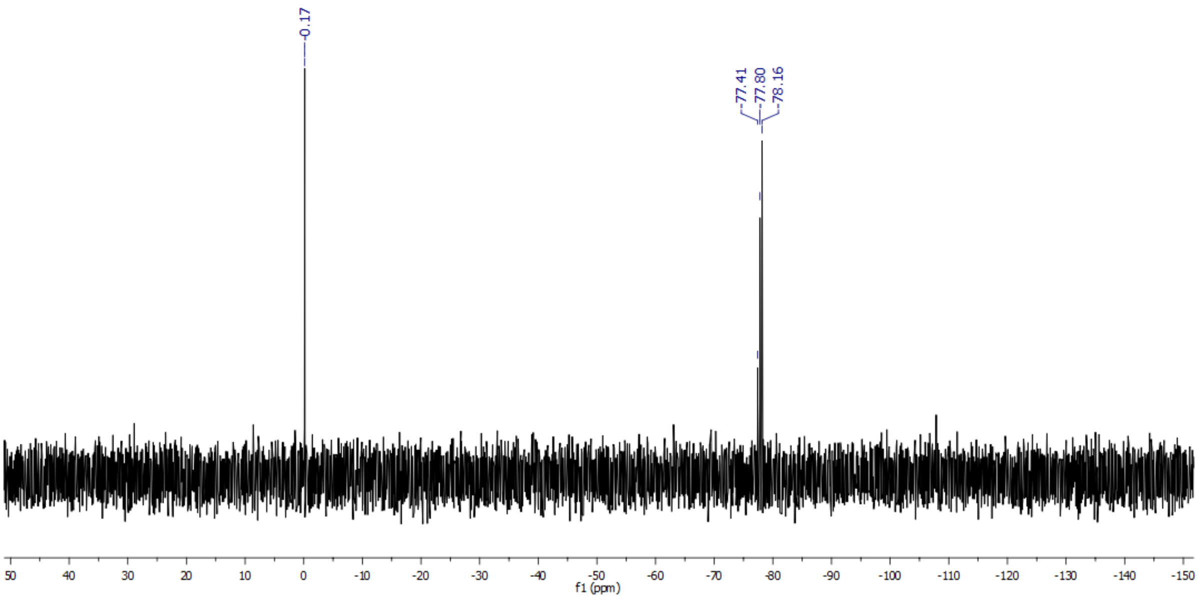

SQ-iBu-All

3,7,14-tris[(allyl)dimethylsiloxy]-1,3,5,7,9,11,14-hepta(isobutyl)tricyclo[7.3.3^{15,11}]heptasiloxane

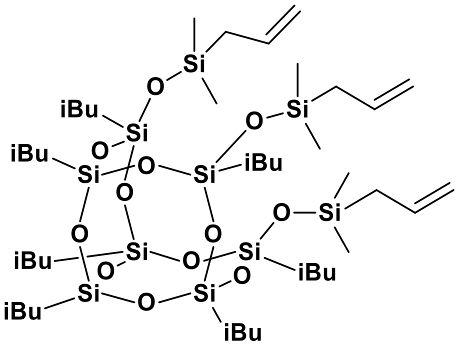

Colorless waxy, isolated yield **94%**

^{1}H NMR (300.2 MHz, $CDCl_3$, δ, ppm): 0.14 (s, 18H, -SiC***H_3***), 0.53-0.58 (m, 14H, -C***H_2***- (iBu)), 0.95-0.98 (m, 42H, -C***H_3*** (iBu)), 1.61 (dd, $J_{H,H}$ = 8.1 Hz, 6H, H_2C=CH-C***H_2***-), 1.79-1.89 (m, 7H, -C***H***-(iBu)), 4.82-4.89 (m, 6H, ***H_2***C=CH-), 5.72-5.86 (m, 3H, H_2C=C***H***-). **^{13}C NMR** (100.6 MHz, $CDCl_3$, δ, ppm): -0.09 (-Si**C**H_3), 22.59, 23.90, 24.14-24.23, 25.12, 25.78, 26.01-26.18 (iBu), 26.27 (H_2C=CH-**C**H_2-), 113.49 (H_2**C**=CH-), 134.53 (H_2C=**C**H-). **^{29}Si NMR** (79.5 MHz, $CDCl_3$, δ, ppm): 5.92 (-O***Si***$(CH_3)_2$-), -67.30, -67.63, -68.02.

FT-IR (cm^{-1}): 3078.71 (=C-H), 2953.89, 2870.35 (-C-H), 1631.41 (C=C), 1465.62 (-C-H), 1253.53 (Si-C), 1075.85, 1047.24 (Si-O).

Chemical formula: $C_{43}H_{96}O_{12}Si_{10}$. **MALDI-ToF MS:** Calcd. for $C_{43}H_{96}H^{+}Na^{+}O_{12}Si_{10}$: *m/z* 1108.4559, found: 1108.4549.

^{1}H NMR

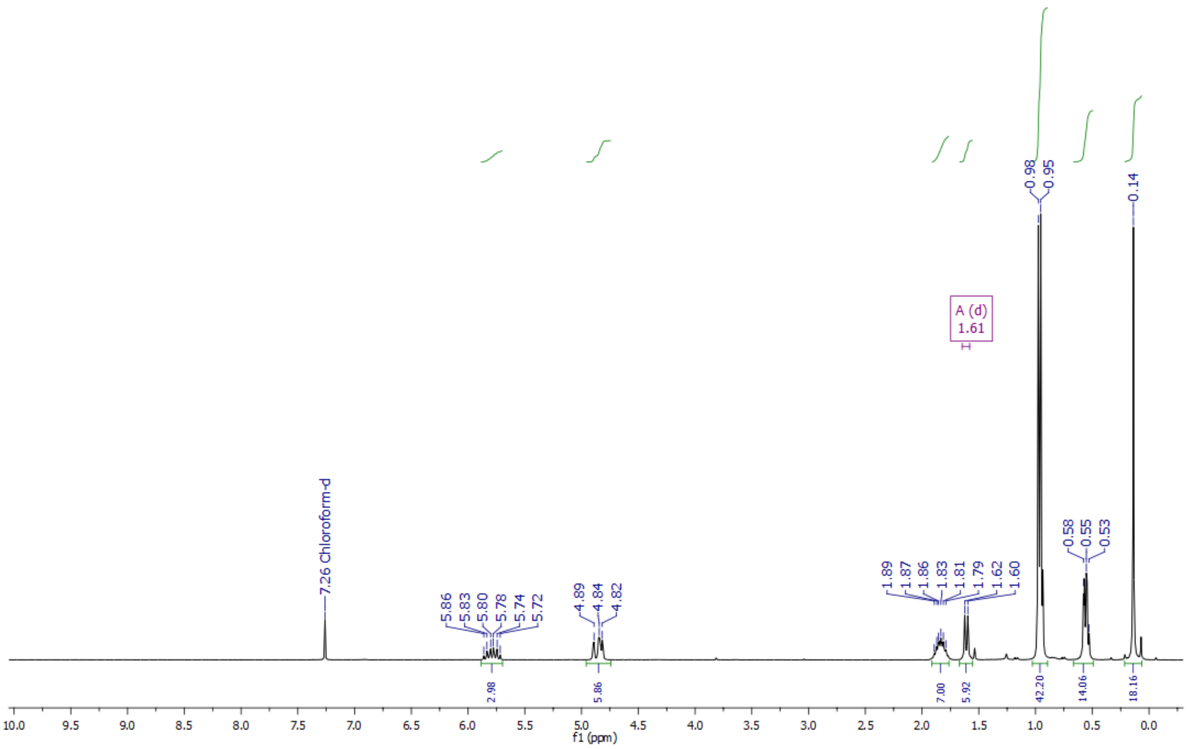

^{13}C NMR

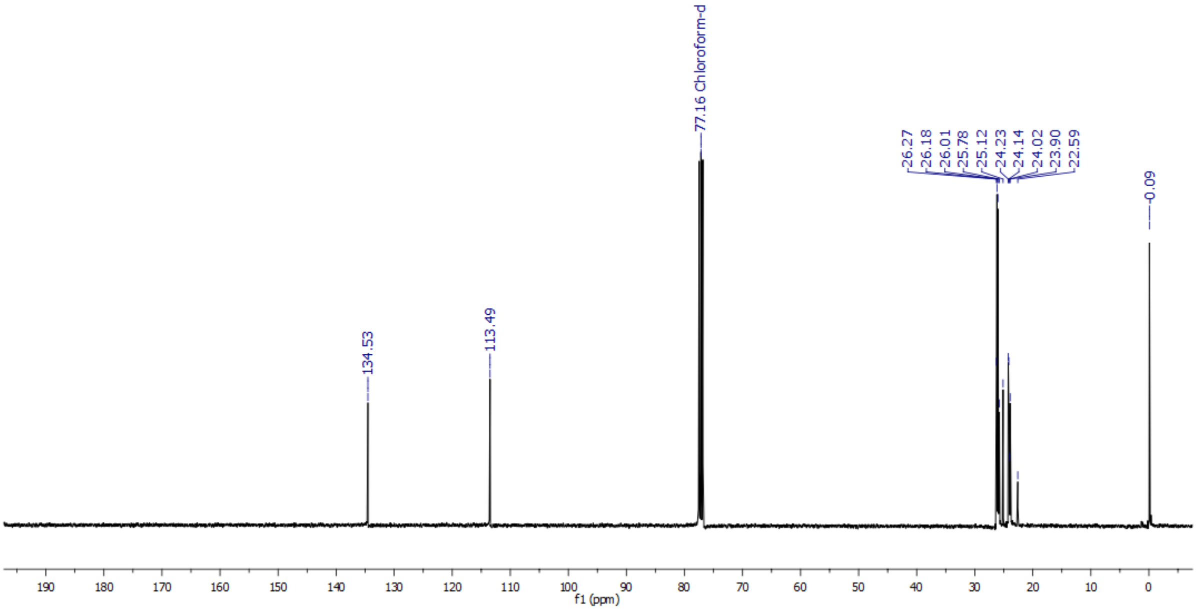

^{29}Si NMR

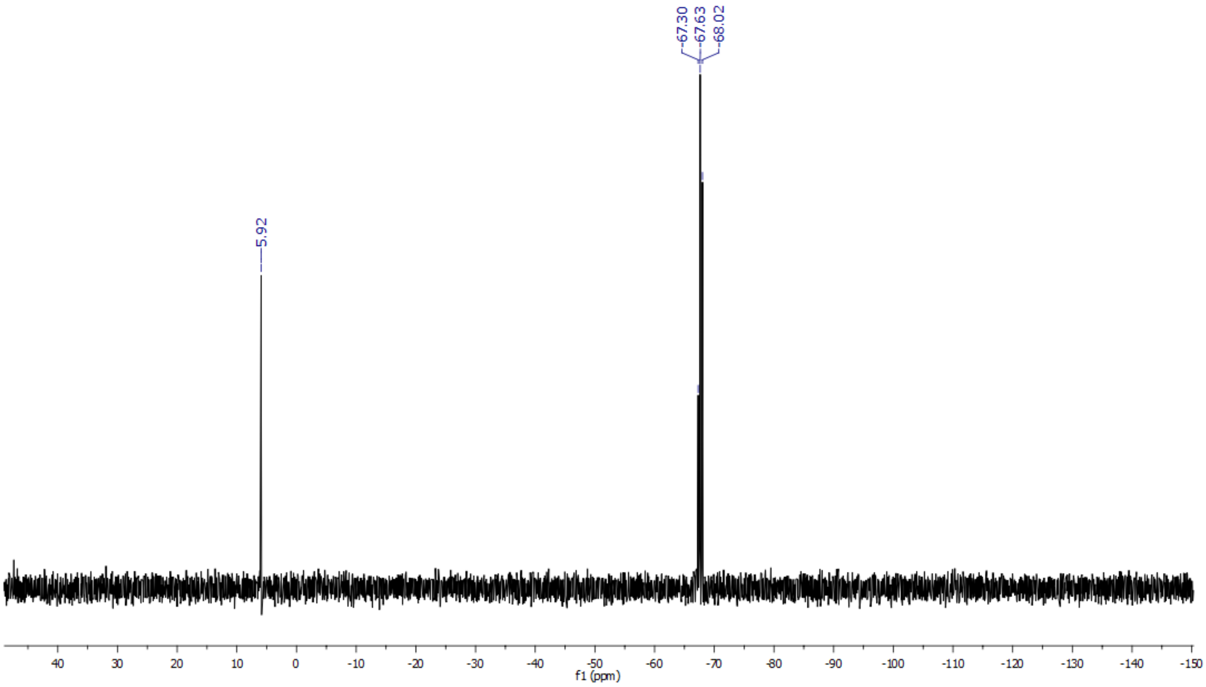

SQ-Ph-All

3,7,14-tris[(allyl)dimethylsiloxy]-1,3,5,7,9,11,14-hepta(phenyl)tricyclo[7.3.3^{15,11}]heptasiloxane

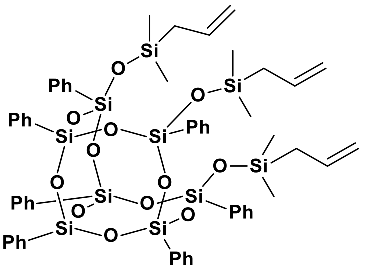

White solid, isolated yield **92%**
^{1}H NMR (300.2 MHz, $CDCl_3$, δ, ppm): 0.26 (s, 18H, -SiC***H₃***), 1.70 (dd, $J_{H,H}$ = 7.9 Hz, 6H, H_2C=CH-C***H₂***-), 4.83-4.91 (m, 6H, ***H***$_2$C=CH-), 5.74-5.88 (m, 3H, H_2C=C***H***-), 7.08-7.57 (m, 35H, **Ph**). **^{13}C NMR** (100.6 MHz, $CDCl_3$, δ, ppm): -0.06 (-Si**C**H_3), 26.15 (H_2C=CH-**C**H_2-), 113.91 (H_2**C**=CH-), 127.64-127.71, 128.02, 130.14-130.28, 131.21, 132.80 (Ph), 134.09 (H_2C=**C**H-), 134.14-134.16 (Ph). **^{29}Si NMR** (79.5 MHz, $CDCl_3$, δ, ppm): 8.53 (-O***Si***$(CH_3)_2$-), -77.41, -77.81, -78.04.
FT-IR (cm^{-1}): 3073.09, 3027.62 (C-H phenyl), 2958.51, 2916.05 (-C-H), 1630.24 (C=C), 1594.32 (C=C phenyl), 1489.96 (-C-H), 1430.03 (C=C phenyl), 1255.44 (Si-C), 1129.05, 1047.24 (Si-O), 997.89 (C-H phenyl).
Chemical formula: $C_{57}H_{68}O_{12}Si_{10}$. **MALDI-ToF MS:** Calcd. for $C_{57}H_{68}HNaO_{12}Si_{10}$: *m/z* 1248.2379, found: 1248.2390.

^{1}H NMR

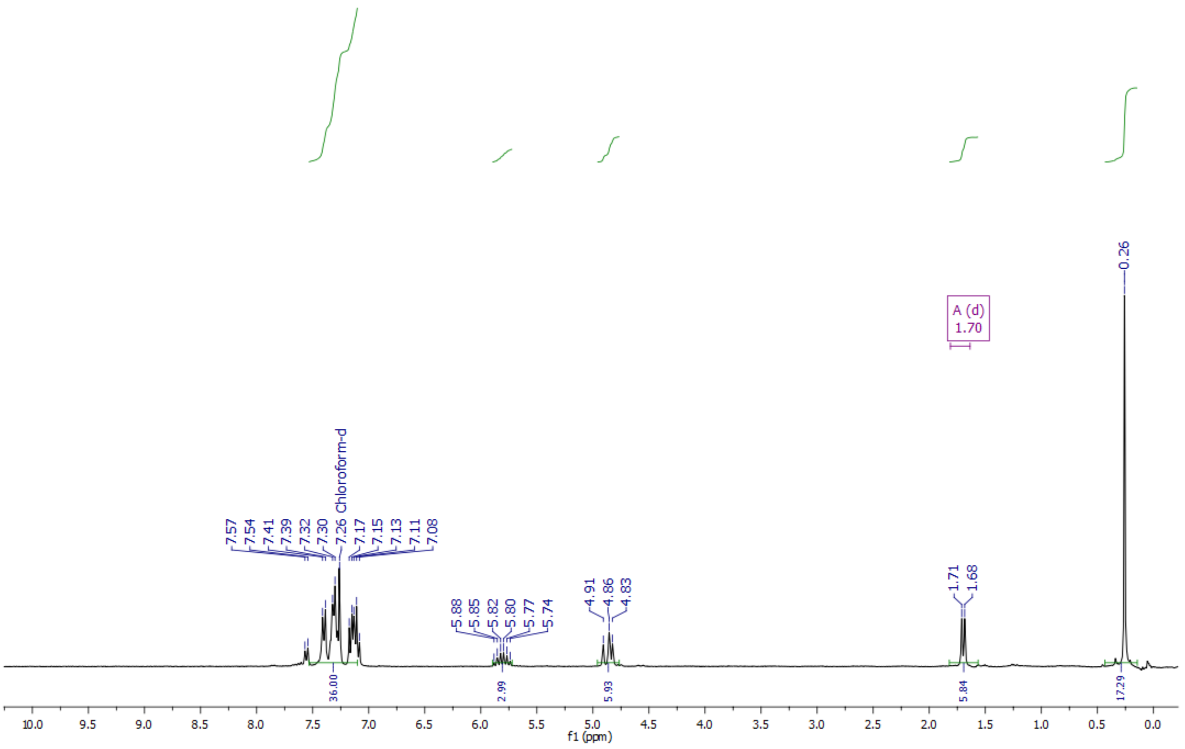

^{13}C NMR

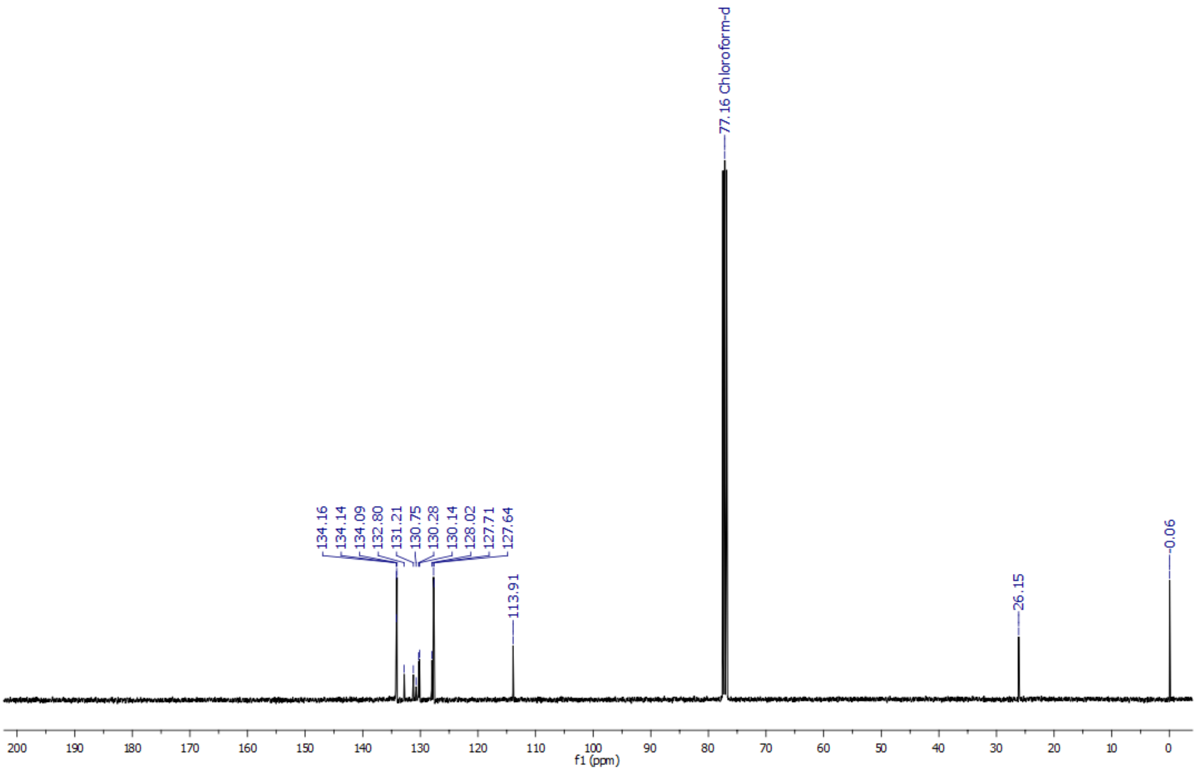

^{29}Si NMR

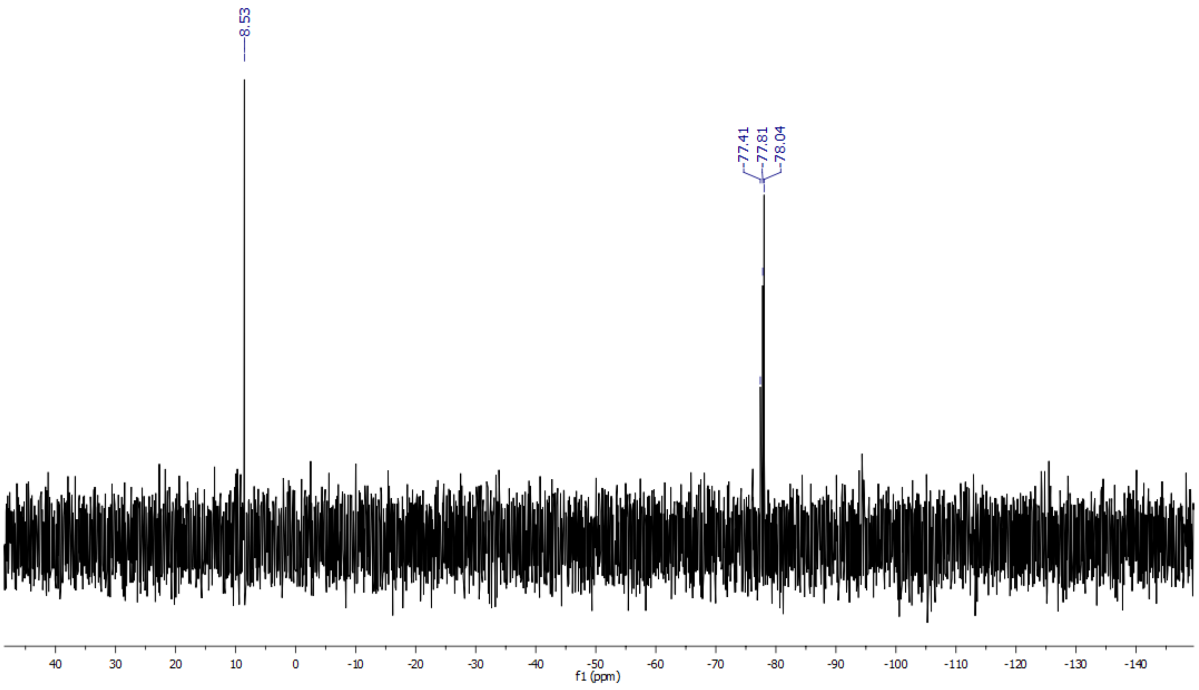

SQ-iBu-Hex

3,7,14-tris[dimethyl(hex-5-enyl)siloxy]-1,3,5,7,9,11,14-hepta(isobutyl)tricyclo[7.3.3^{15,11}]heptasiloxane

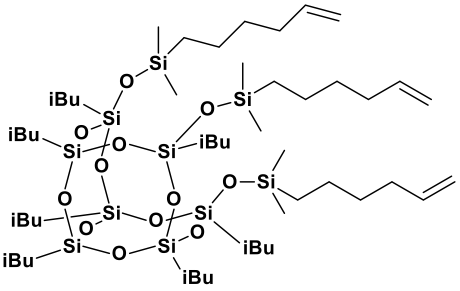

Colorless oil, isolated yield **90%**

^{1}H NMR (300.2 MHz, $CDCl_3$, δ, ppm): 0.11 (s, 18H, -SiC$\boldsymbol{H_3}$), 0.53-0.59 (m, 20H, -C$\boldsymbol{H_2}$- (iBu, hexenyl)), 0.93-0.97 (m, 42H, -C$\boldsymbol{H_3}$ (iBu)), 1.29-1.42 (m, 12H, -C$\boldsymbol{H_2}$- (hexenyl)), 1.81-1.87 (m, 7H, -C***H***- (iBu)), 2.00-2.07 (m, 6H, -C$\boldsymbol{H_2}$- (hexenyl)), 4.90-5.01 (m, 6H, $\boldsymbol{H_2}$C=CH-), 5.74-5.88 (m, 3H, H_2C=C***H***-). **^{13}C NMR** (100.6 MHz, $CDCl_3$, δ, ppm): 0.47 (-Si**C**H_3), 18.14 (-**C**H_2- (hexenyl)), 22.62 (iBu), 22.62 (-**C**H_2- (hexenyl)), 23.94, 24.02, 24.14, 24.25, 25.21, 25.79, 26.02, 26.19 (iBu), 114.19 (H_2**C**=CH-), 139.33 (H_2C=**C**H-). **^{29}Si NMR** (79.5 MHz, $CDCl_3$, δ, ppm): 9.02 (-O***Si***$(CH_3)_2$-), -67.35, -67.78, -68.23.

FT-IR (cm^{-1}): 3076.41 (=C-H), 2952.88, 2868.19 (-C-H), 1641.18 (C=C), 1465.38 (-C-H), 1251.92 (Si-C), 1079.28, 1049.03 (Si-O).

Chemical formula: $C_{52}H_{114}O_{12}Si_{10}$. **MALDI-ToF MS:** Calcd. for $C_{52}H_{114}$ $H^+Na^+O_{12}Si_{10}$: *m/z* 1234.5968, found:1234.5964.

^{1}H NMR

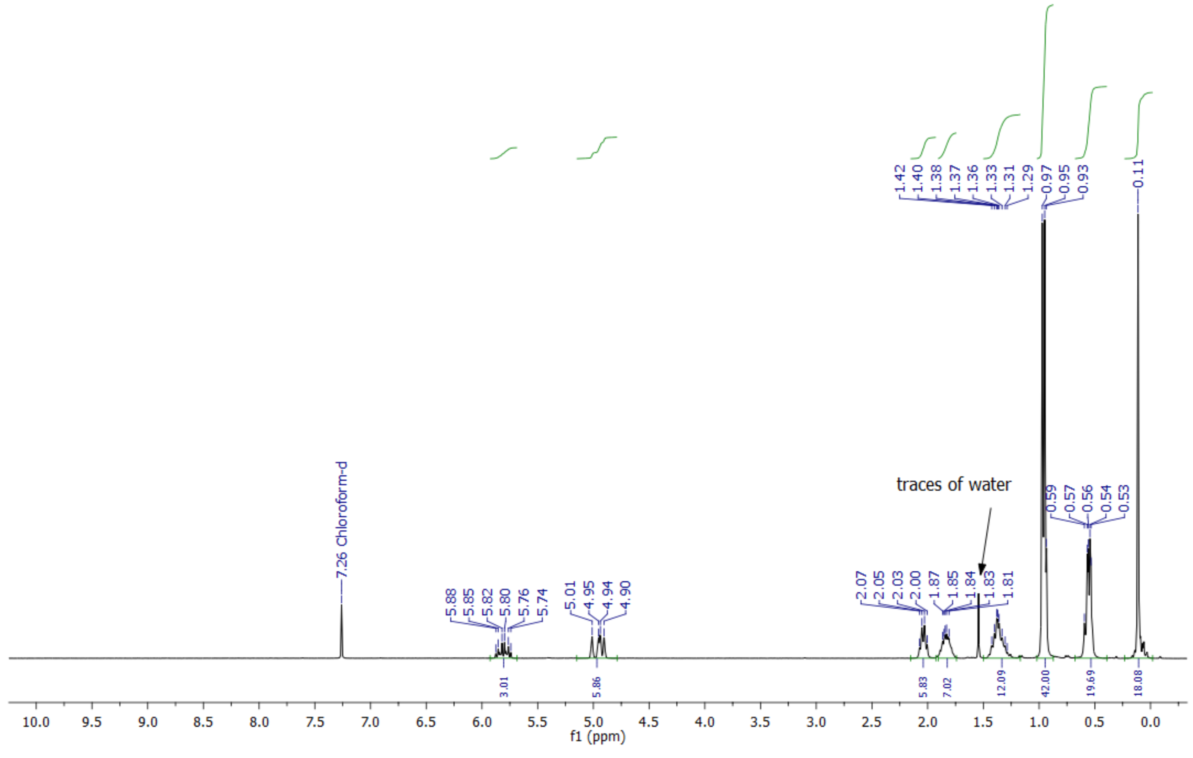

^{13}C NMR

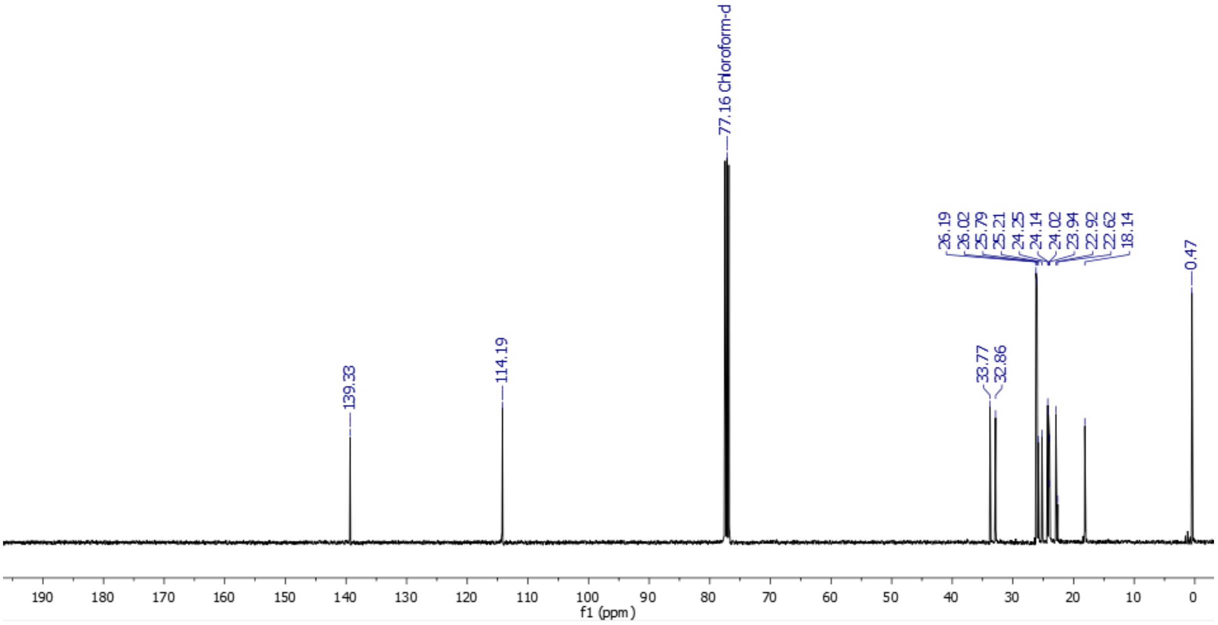

^{29}Si NMR

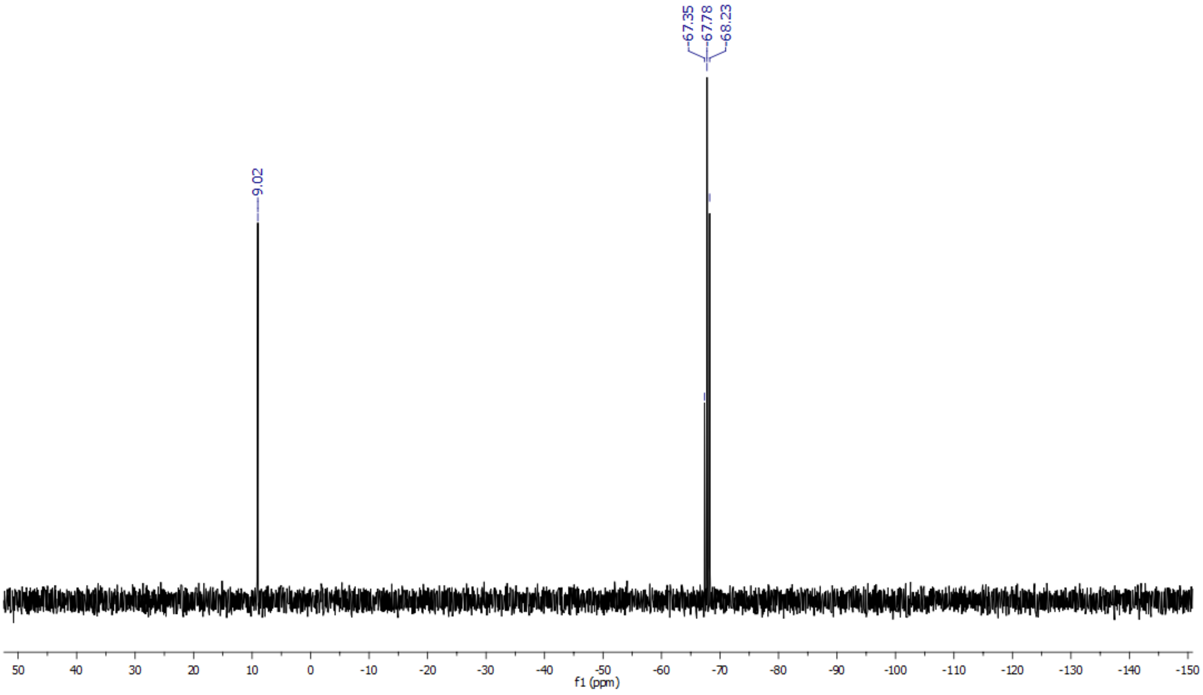

SQ-Ph-Hex

3,7,14-tris[dimethyl(hex-5-enyl)siloxy]-1,3,5,7,9,11,14-hepta(phenyl)tricyclo[7.3.3^{15,11}]heptasiloxane

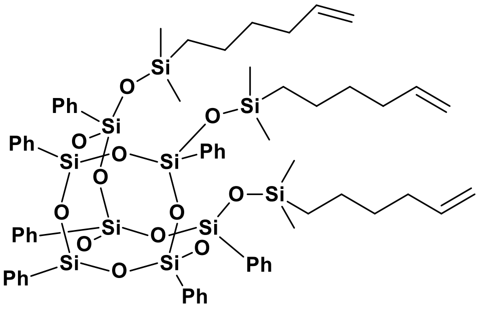

White solid, isolated yield **84%**

^{1}H NMR (300.2 MHz, $CDCl_3$, δ, ppm): 0.25 (s, 18H, -SiC***H*₃**), 0.63-0.68 (m, 6H, -C***H*₂**- (hexenyl)), 1.35-1.38 (m, 12H, -C***H*₂**- (hexenyl)), 1.94-2.01 (m, 6H, -C***H*₂**- (hexenyl)), 4.88-4.97 (m, 6H, ***H*₂**C=CH-), 5.70-5.83 (m, 3H, H_2C=C***H*-**), 7.09-7.57 (m, 35H, **Ph**). **^{13}C NMR** (100.6 MHz, $CDCl_3$, δ, ppm): -0.52 (-Si**C**H_3), 18.13, 22.87, 32.83, 33.68 (-**C**H_2- (hexenyl)), 114.20 (H_2**C**=CH-), 127.61-127.68, 127.99, 130.04-130.23, 130.70, 131.38, 133.10, 134.10-134.17 (Ph), 139.26 (H_2C=**C**H-). **^{29}Si NMR** (79.5 MHz, $CDCl_3$, δ, ppm): 11.78 (-O***Si***(CH_3)$_2$-), -77.38, -77.90, -78.12.

FT-IR (cm^{-1}): 3072.75, 3051.80 (C-H phenyl), 2955.76, 2853.79 (-C-H), 1640.29 (C=C), 1593.88 (C=C phenyl), 1489.77 (-C-H), 1429.86 (C=C phenyl), 1251.59 (Si-C), 1045.76 (Si-O), 997.84 (C-H phenyl).

Chemical formula: $C_{66}H_{86}O_{12}Si_{10}$. **MALDI-ToF MS:** Calcd. for $C_{66}H_{86}HNa^+O_{12}Si_{10}$: *m/z* 1374.3782, found: 1374.3783.

^{1}H NMR

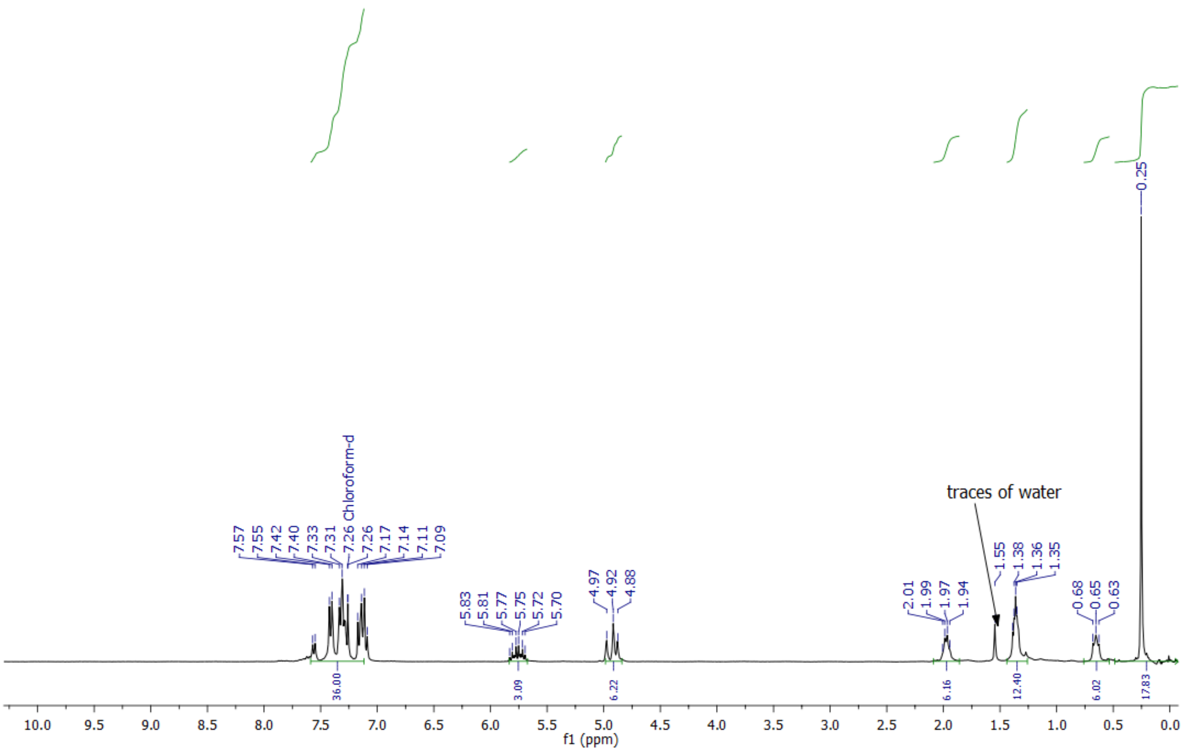

^{13}C NMR

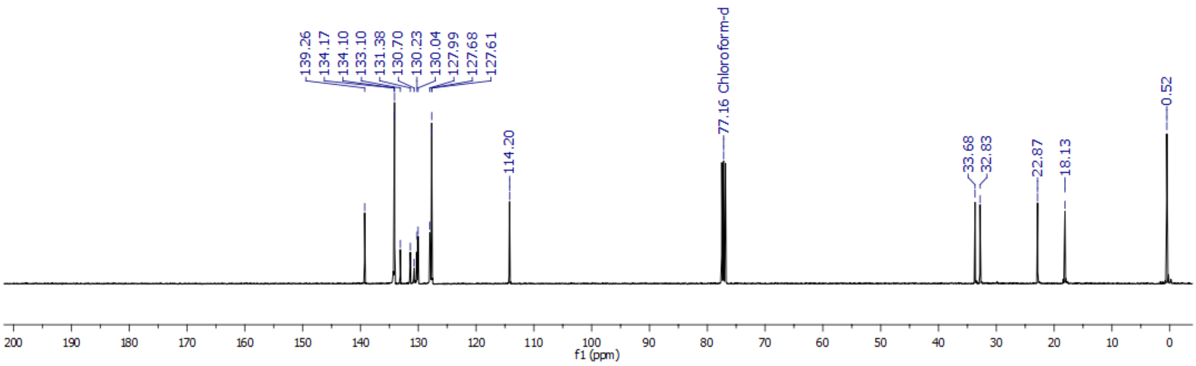

^{29}Si NMR

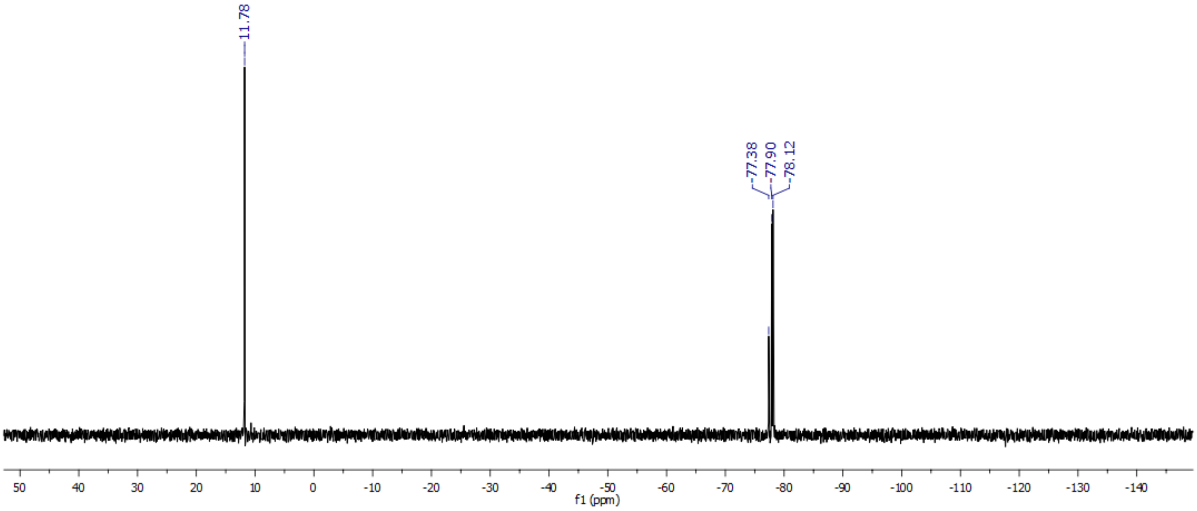

SQ-iBu-Dec

3,7,14-tris[dimethyl(dec-9-enyl)siloxy]-1,3,5,7,9,11,14-hepta(isobutyl)tricyclo[7.3.3^{15,11}]heptasiloxane

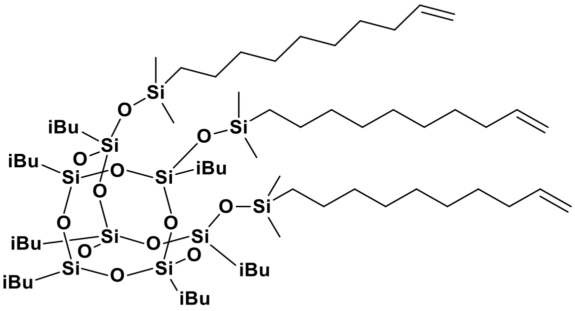

Colorless oil, isolated yield **89%**

^{1}H NMR (300.2 MHz, $CDCl_3$, δ, ppm): 0.11 (s, 18H, -SiC***H*₃**), 0.53-0.56 (m, 20H, -C***H*₂**- (iBu, decenyl)), 0.93-0.97 (m, 42H, -C***H*₃** (iBu)), 1.28-1.37 (m, 36H, -C***H*₂**- (decenyl)) 1.79-1.89 (m, 7H, -C***H***- (iBu)), 2.00-2.07 (m, 6H, -C***H*₂**- (decenyl)), 4.91-5.01 (m, 6H, ***H*₂**C=CH-), 5.75-5.88 (m, 3H, H_2C=C***H***-). **^{13}C NMR** (100.6 MHz, $CDCl_3$, δ, ppm): 0.48 (-Si**C**H_3), 18.31, 23.38 (-**C**H_2- (decenyl)), 23.94-24.25, 25.21, 25.78, 26.03, 26.19 (iBu), 29.14, 29.41, 29.59, 29.68, 33.69, 34.00 (-**C**H_2- (decenyl)), 114.23 (H_2**C**=CH-), 139.42 (H_2C=**C**H-). **^{29}Si NMR** (79.5 MHz, $CDCl_3$, δ, ppm): 9.09 (-O***Si***$(CH_3)_2$-), -67.35, -67.82, -68.30.

FT-IR (cm^{-1}): 3076.99 (=C-H), 2952.57, 2853.82 (-C-H), 1641.10 (C=C), 1465.20 (-C-H), 1251.13 (Si-C), 1074.40 (Si-O).

Chemical formula: $C_{64}H_{138}O_{12}Si_{10}$. **MALDI-ToF MS:** Calcd. for $C_{64}H_{138}NaO_{12}Si_{10}$: *m/z* 1401.7779, found: 1401.7794.

^{1}H NMR

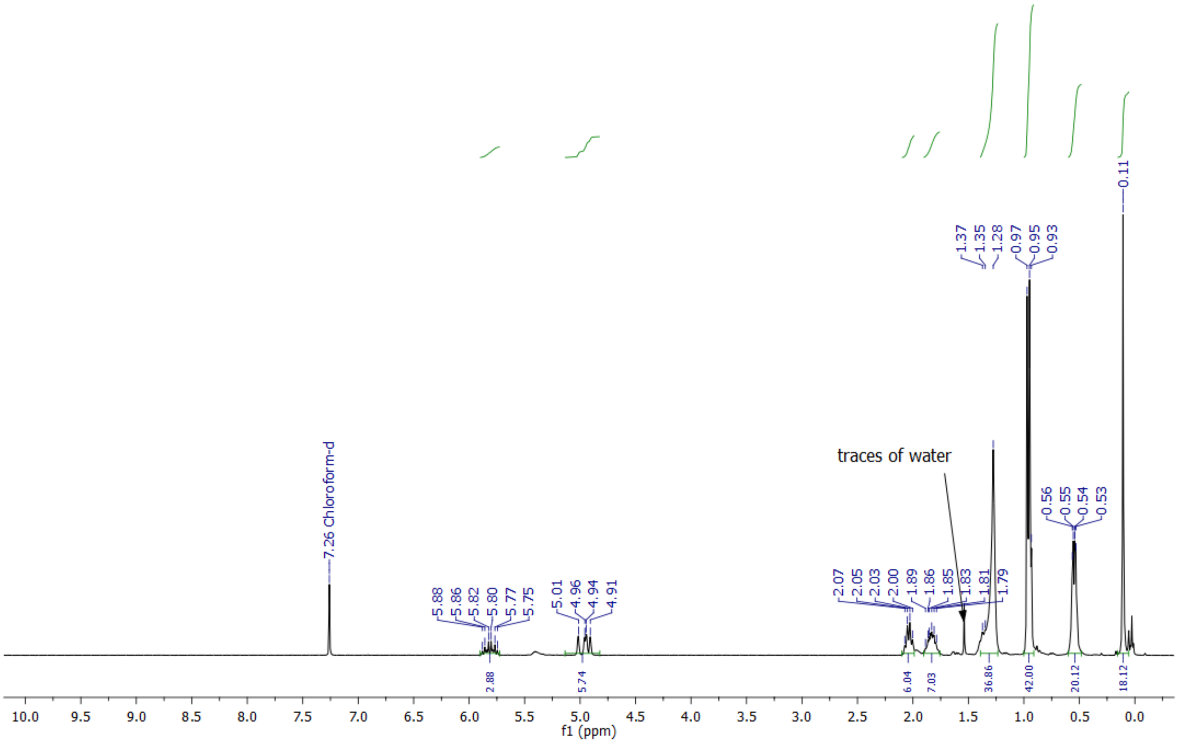

^{13}C NMR

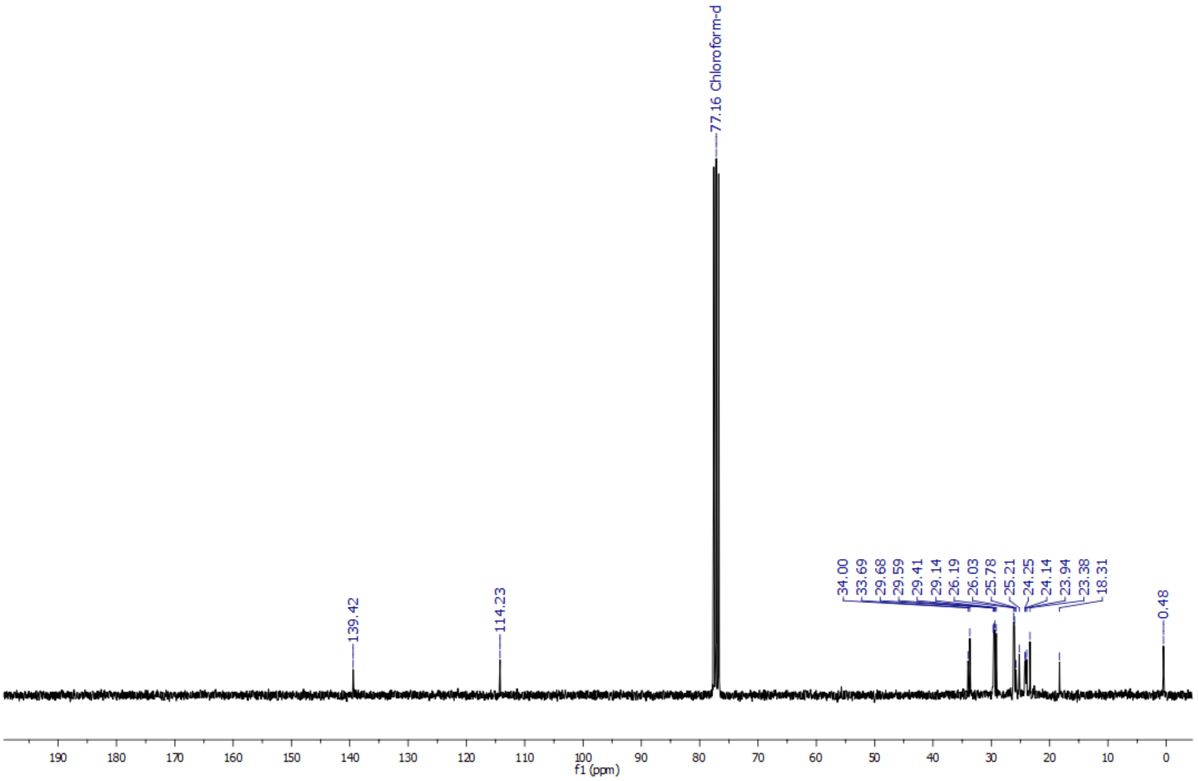

^{29}Si NMR

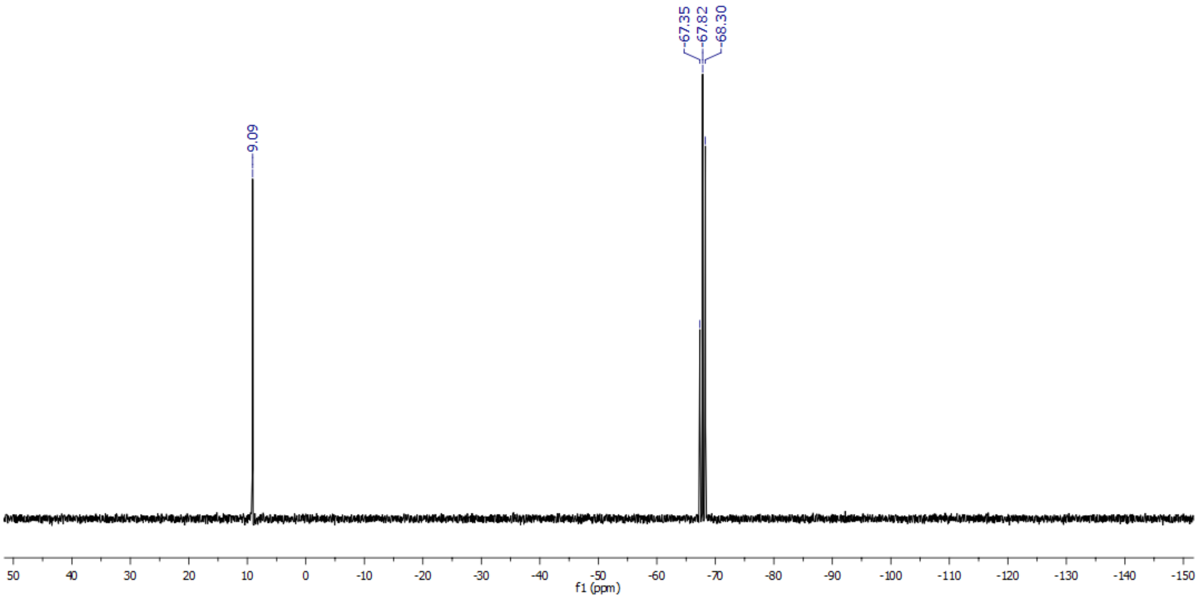

SQ-Ph-Dec

3,7,14-tris[dimethyl(dec-9-enyl)siloxy]-1,3,5,7,9,11,14-hepta(phenyl)tricyclo[7.3.3^{15,11}]heptasiloxane

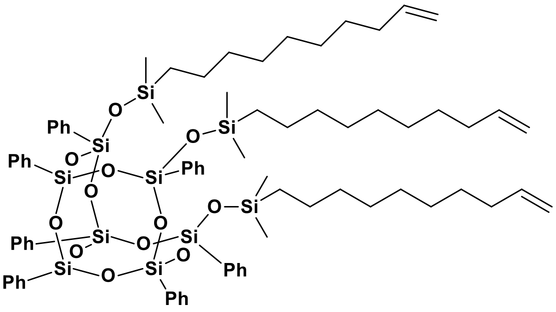

White solid, isolated yield **85%**

^{1}H NMR (300.2 MHz, $CDCl_3$, δ, ppm): 0.24 (s, 18H, -SiC***H***$_3$), 0.61-0.66 (m, 6H, -C***H*$_2$**- (decenyl)), 1.21-1.32 (m, 36H, -C***H*$_2$**- (decenyl)), 1.99-2.05 (m, 6H, -C***H*$_2$**- (decenyl)), 4.90-5.02 (m, 6H, ***H*$_2$**C=CH-), 5.74-5.87 (m, 3H, H_2C=C***H*-**), 7.07-7.56 (m, 35H, **Ph**). **^{13}C NMR** (100.6 MHz, $CDCl_3$, δ, ppm): 0.55 (-Si**C**H_3), 18.32, 23.36, 29.14, 29.40, 29.54, 29.67, 33.69, 34.02 (-**C**H_2- (decenyl)), 114.26 (H_2**C**=CH-), 127.60-127.67, 128.00, 130.02-130.20, 130.70, 131.43, 133.18, 134.12-134.19 (Ph), 139.37 (H_2C=**CH-**). **^{29}Si NMR** (79.5 MHz, $CDCl_3$, δ, ppm): 11.88 (-O***Si***$(CH_3)_2$-), -77.35, -77.94, -78.12.

FT-IR (cm^{-1}): 3072.82, 3005.99 (C-H phenyl), 2922.05, 2852.58 (-C-H), 1640.29 (C=C), 1594.23 (C=C phenyl), 1489.92 (-C-H), 1429.92 (C=C phenyl), 1251.05 (Si-C), 1129.45, 1048.09 (Si-O), 997.84 (C-H phenyl).

Chemical formula: $C_{78}H_{110}O_{12}Si_{10}$. **MALDI-ToF MS:** Calcd. for $C_{78}H_{110}$ $HNa^{+}O_{12}Si_{10}$: *m/z* 1541.5488, found: 1541.5588.

^{1}H NMR

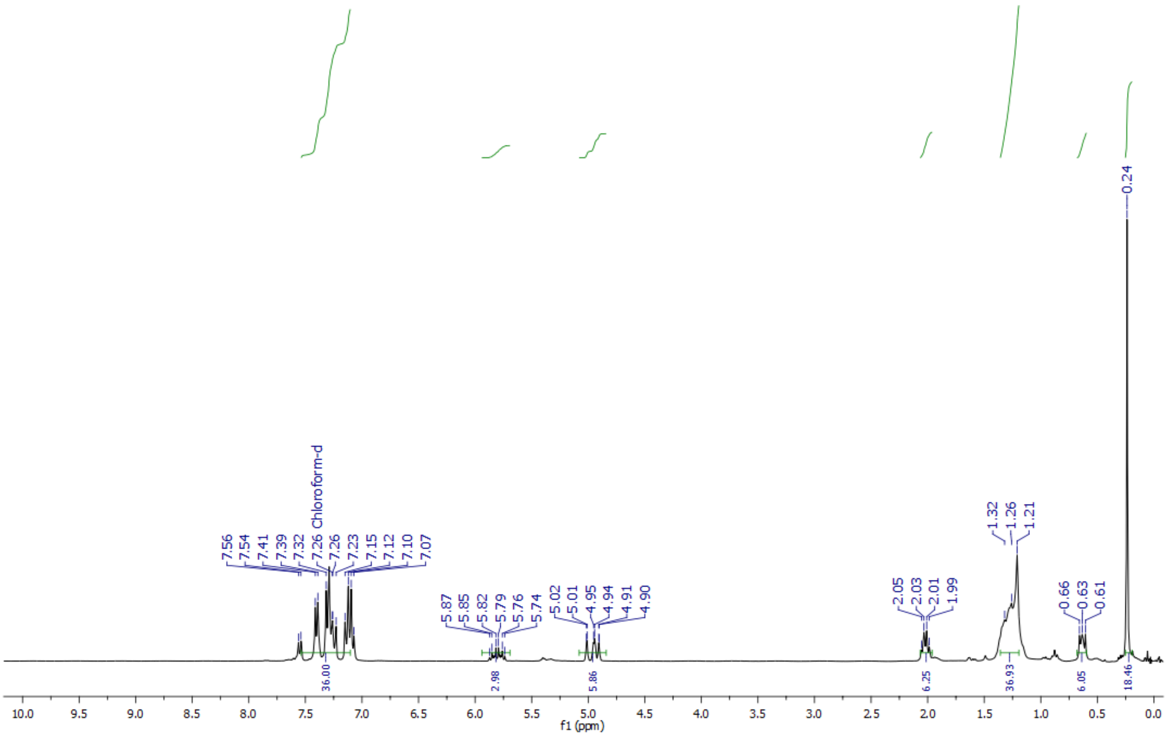

^{13}C NMR

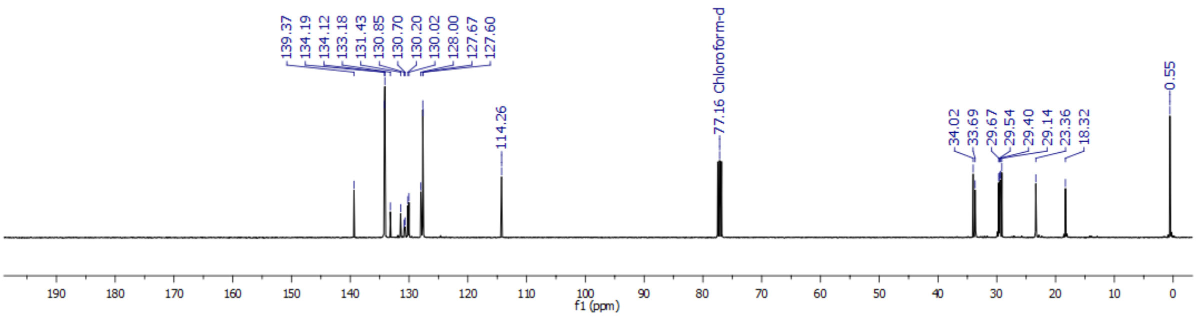

^{29}Si NMR

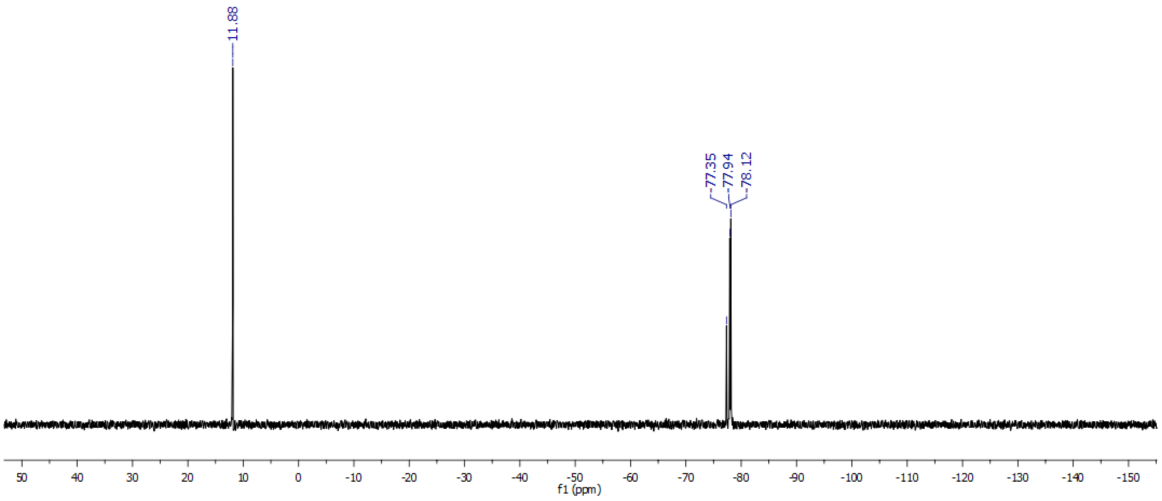

2. Additional NMR spectra

2.1 Product SQ-iBu-Hex obtained in a different conditions of condensation reaction

a) in a concentrated THF solution (0.63M) – a complete conversion of **SQ-iBu-OH** is observed.

b) diluted THF solution (0.13M) – an incomplete condensation of **SQ-iBu-OH** and presence of Si-OH moiety at -58 ppm is noted.

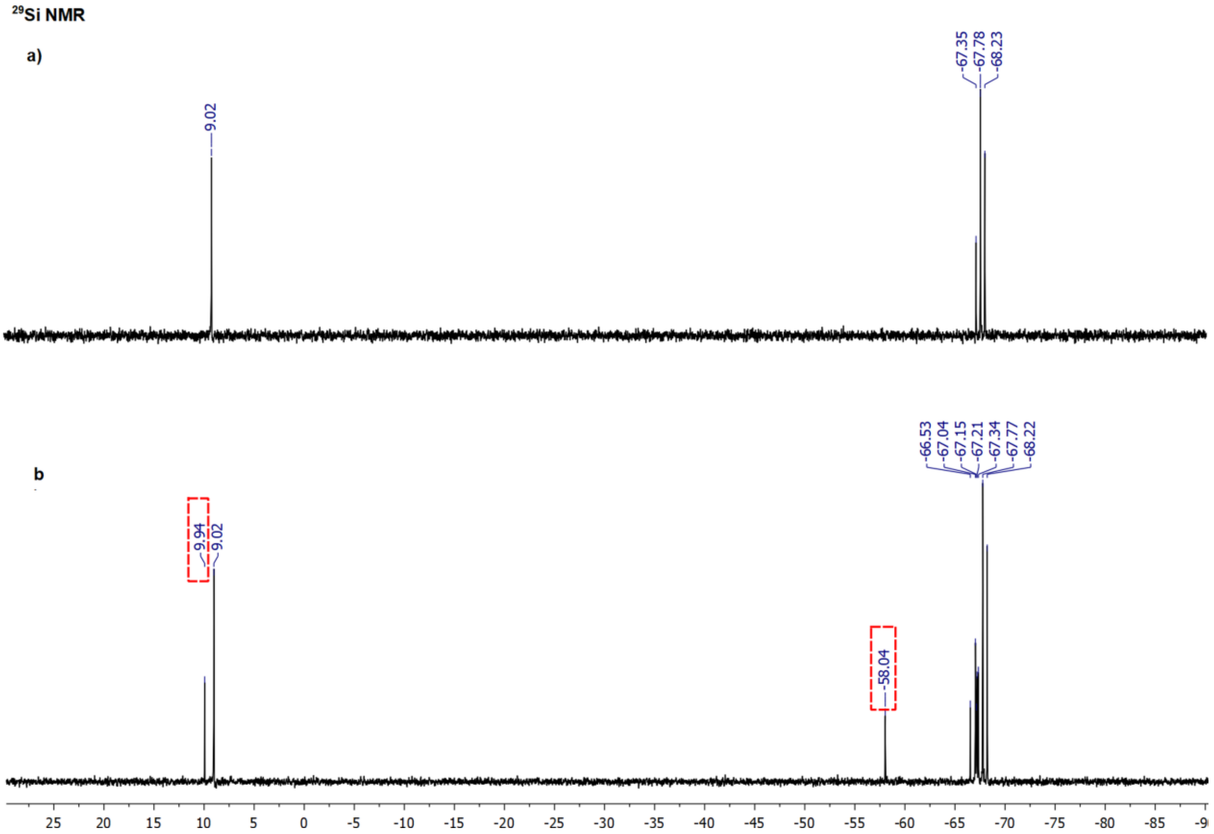

Figure S1. ^{29}Si NMR spectra of isolated **SQ-iBu-Hex** obtained *via* condensation reaction performed a) in a concentrated THF solution (0.63M); b) diluted THF solution (0.13M)

2.2 Thermal cross-condensation of SQ-iBu-Vi

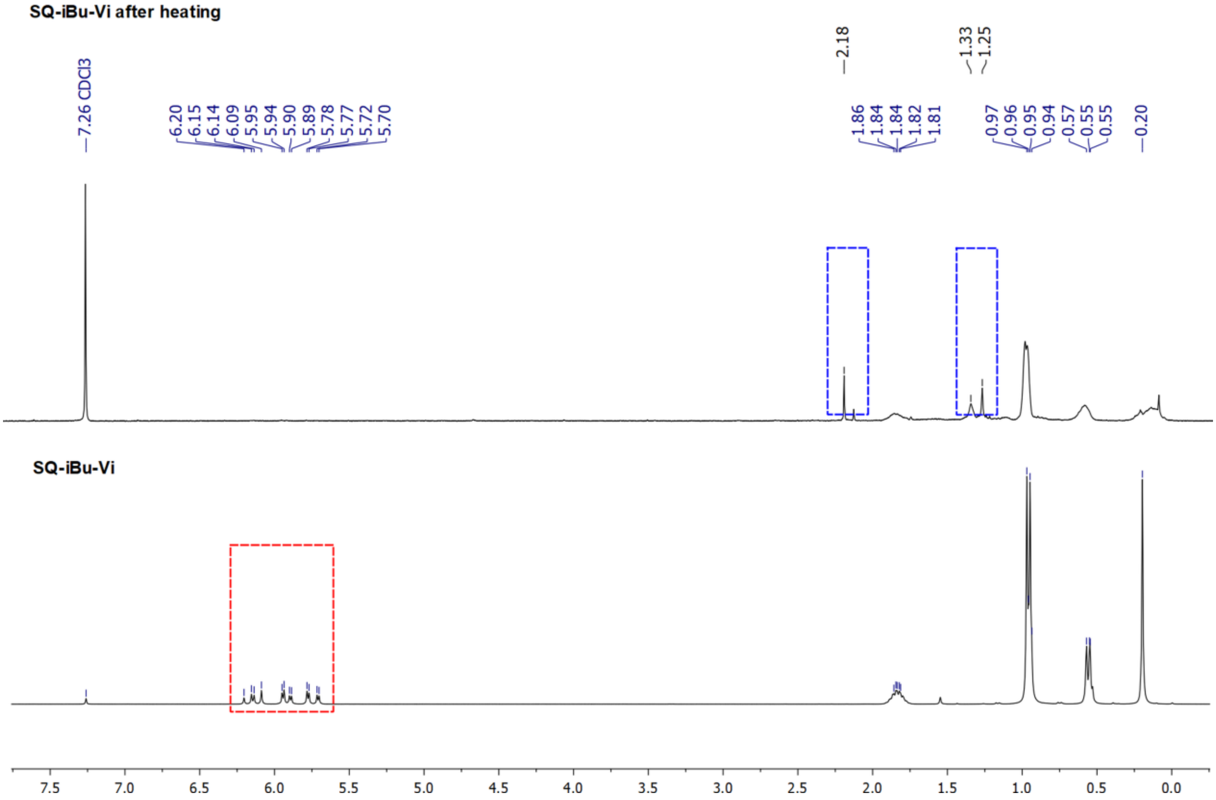

Figure S2. ^{1}H NMR spectra of isolated **SQ-iBu-Vi** and material abbreviated as **SQ-iBu-Vi after heating**, obtained as a result of 30 min heating of neat **SQ-iBu-Vi** at 200°C at steel plate.

3. Products of hydrosilylation of dienes by SQ-R-SiH conducted in different conditions

a) Hydrosilylation of 1,5-hexadiene/1,9-decadiene by SQ-R-SiH in a diluted toluene solution (0.04M)

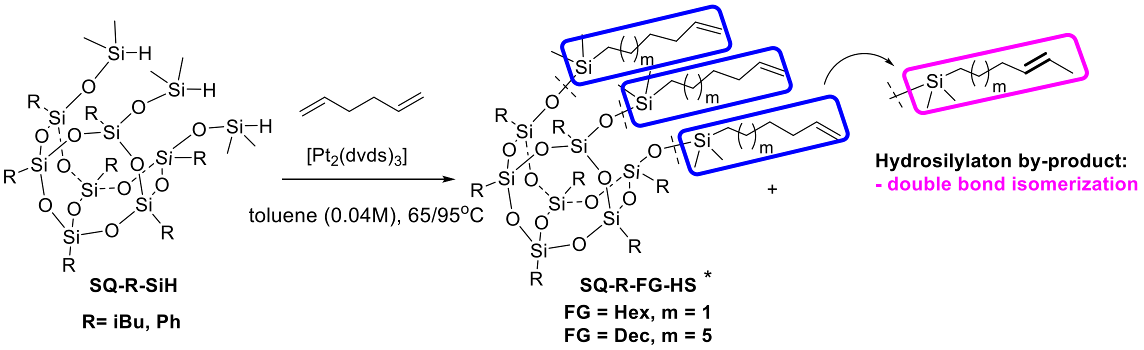

* accompanied by compounds of alkenyl chains with isomerized C=C group

b) Hydrosilylation of 1,5-hexadiene by SQ-R-SiH in a concentrated toluene solution (0.22-0.25M) and one of possible structures of an aggregate

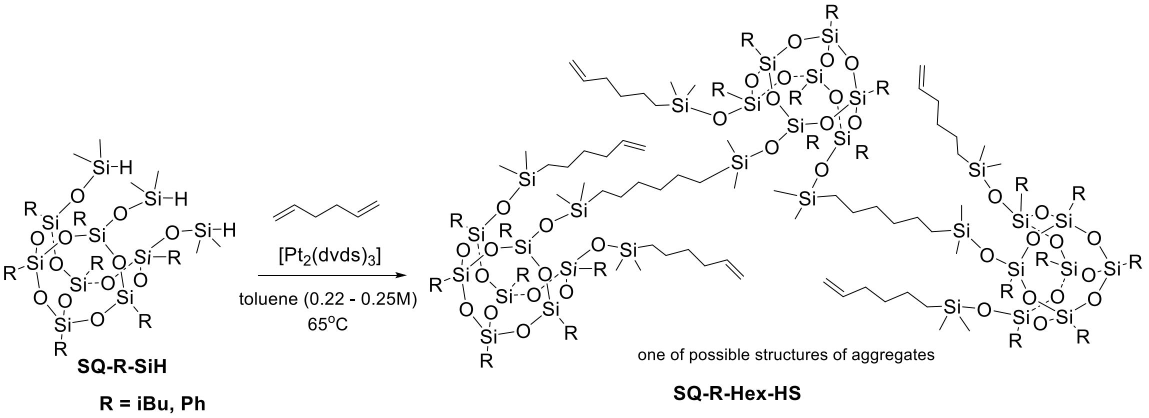

4. GPC chromatograms

Hydrosilylation of 1,5-hexadiene by SQ-R-SiH in a concentrated toluene solution (R=iBu, 0.22M; R=Ph, 0.25M) resulting in **SQ-R-Hex-HS**.

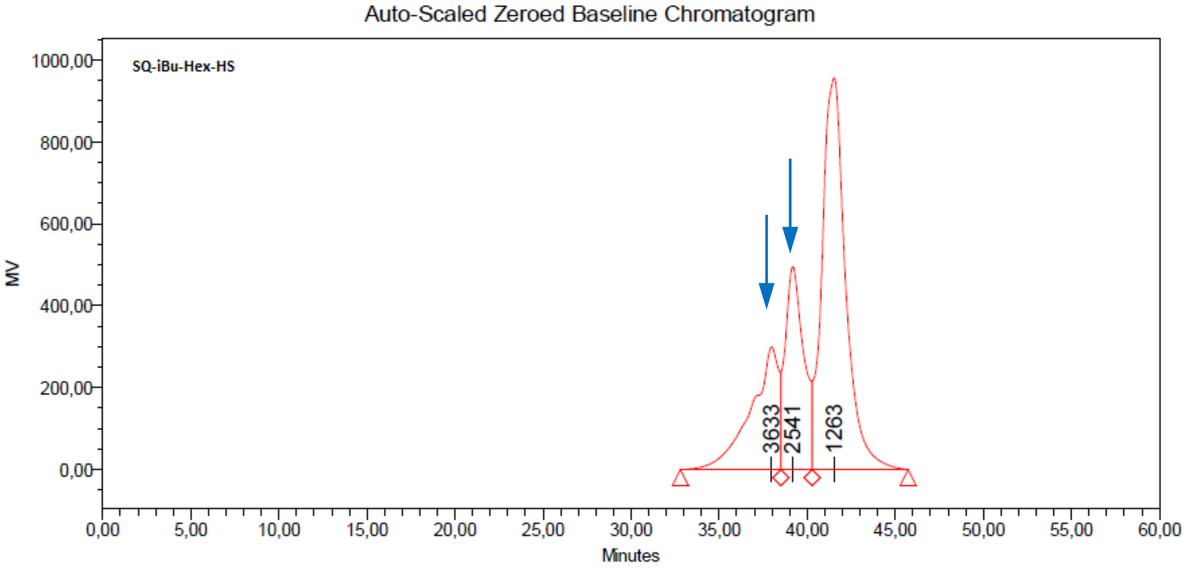

Figure S3. GPC analysis of crude **SQ-iBu-Hex-HS**, obtained *via* hydrosilylation of 1,5-hexadiene by **SQ-iBu-SiH** at 0.22M concentration of **SQ-iBu-SiH** in toluene. The marked peaks correspond with the higher M_w of compounds resulting from intermolecular hydrosilylation of 1,5-hexadiene by two or three **SQ-iBu-SiH** molecules.

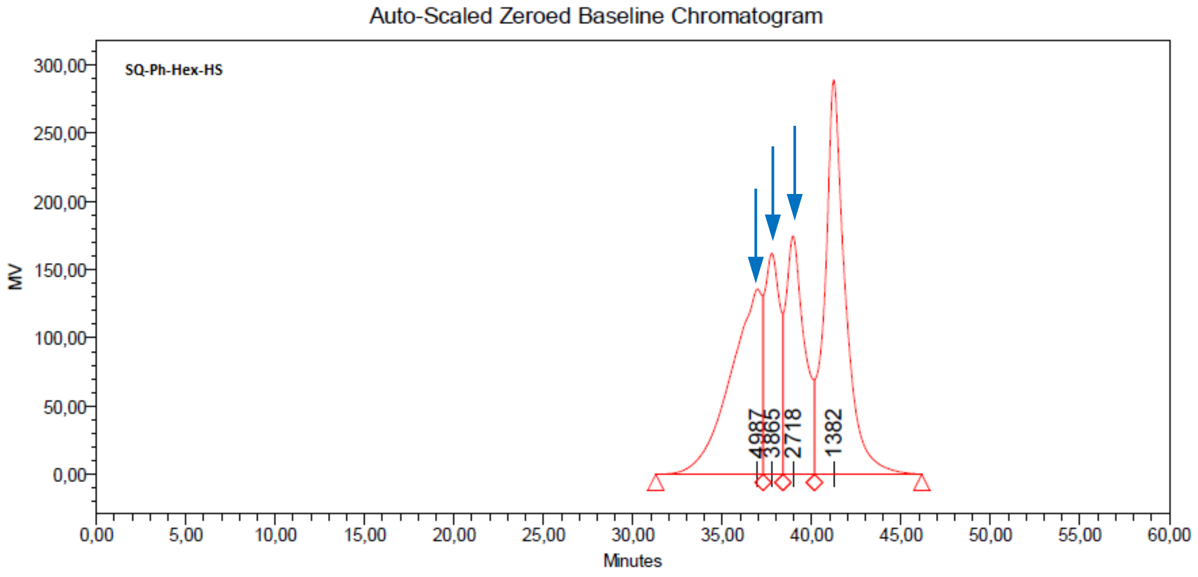

Figure S4. GPC analysis of crude **SQ-Ph-Hex-HS**, obtained *via* hydrosilylation of 1,5-hexadiene by **SQ-Ph-SiH** at 0.22M concentration of **SQ-Ph-SiH** in toluene. The marked peaks correspond with the higher M_w of compounds resulting from intermolecular hydrosilylation of 1,5-hexadiene by two or three **SQ-Ph-SiH** molecules.

5. References

1. Dutkiewicz, M.; Karasiewicz, J.; Rojewska, M.; Skrzypiec, M.; Dopierała, K.; Prochaska, K.; Maciejewski, H. Synthesis of an Open-Cage Structure POSS Containing Various Functional Groups and Their Effect on the Formation and Properties of Langmuir Monolayers. *Chem. - A Eur. J.* **2016**, *22*, 13275–13286.

2. Yuasa, S.; Sato, Y.; Imoto, H.; Naka, K. Fabrication of composite films with poly(methyl methacrylate) and incompletely condensed cage-silsesquioxane fillers. *J. Appl. Polym. Sci.* **2018**, *135*, 46033.

3. Kaźmierczak, J.; Kuciński, K.; Hreczycho, G. Highly Efficient Catalytic Route for the Synthesis of Functionalized Silsesquioxanes. *Inorg. Chem.* **2017**, *56*, 9337–9342.
